# Can circulating cell free DNA be a promising marker in ovarian cancer? – a genome-scale profiling study in a single institution

**DOI:** 10.1186/s13048-022-01068-z

**Published:** 2023-01-14

**Authors:** Huimei Zhou, Xueying Zhang, Qian Liu, Jiaxin Yang, Jian Bai, Min Yin, Dongyan Cao, Qingzheng Zhang, Lu Zheng

**Affiliations:** 1 National Clinical Research Center for Obstetric & Gynecologic DiseasesDepartment of Obstetrics and Gynecology, Peking Union Medical College Hospital, Chinese Academy of Medical Sciences & Peking Union Medical College, Shuaifuyuan, Dongcheng-Qu Beijing, People’s Republic of China; 2 Berry Oncology Corporation, Beijing, China; 3 Fujian Key Laboratory of Advanced Technology for Cancer Screening and Early Diagnosis, Fuzhou, China

## Abstract

**Background:**

Cell-free DNA (cfDNA) is emerging as a potential biomarker for the detection of ovarian cancer (OC). Recently, we reported a method based upon cfDNA whole-genome sequencing data including the nucleosome distribution (nucleosome footprinting NF), terminal signature sequence (motif), DNA fragmentation (fragment), and copy number variation (CNV).In the present study, we explored whether multiomics early screening technology in cfDNA can be applied for early screening of ovarian cancer.

**Methods:**

Fifty-nine patients with OC and 100 healthy controls were included in this prospective study. Cell-free DNA was extracted from plasma and analyzed by low-pass whole-genome sequencing. Genomic features were obtained for all samples of the cohort, including copy number variation (CNV), 5’-end motifs, fragmentation profiles, and nucleosome footprinting (NF). An integrated scoring system termed the OC score was developed based on the performance of these four features.

**Results:**

All four features showed diagnostic potential for OC. Based on the unique genome features of cfDNA, the OC score has high accuracy in distinguishing OC patients from healthy controls (AUC 97.7%; sensitivity 94.7%; specificity 98.0%) as a new comprehensive diagnostic method for OC. The OC score showed a gradual trend from healthy controls to OC patients with different stages, especially for early OC monitoring of concern, which achieved a satisfactory sensitivity (85.7%) at a high specificity.

**Conclusions:**

This is the first study evaluating the potential of cell-free DNA for the diagnosis of primary OC using multidimensional early screening technology. We present a promising method to increase the accuracy of prediction in patients with OC.

**Supplementary Information:**

The online version contains supplementary material available at 10.1186/s13048-022-01068-z.

## Background

Ovarian cancer (OC) is a common malignant tumor of female reproductive organs. The mortality rate of OC is higher than that of breast cancer, cervical cancer, and endometrial cancer, ranking first among gynecological malignancies. Approximately 300,000 new cases of OC and 180,000 deaths occur annually worldwide [1] and due to deep ossification in the pelvis of the ovary and a lack of typical clinical symptoms, approximately 75% of patients are diagnosed at advanced stages (stage III/IV). The 5-year survival rate of early-stage patients can reach more than 70%, whereas that of advanced-stage patients is less than 30%. Therefore, the development of markers for early diagnosis and precise treatment of OC is critical to prolonging patient survival.

Current early screening methods for OC mainly include traditional detection and new liquid biopsy. However, traditional tumor screening methods have some technical limitations, such as,the diagnostic sensitivity and specificity of CA125 for OC are low (the sensitivity and specificity are 79% and 78% [2]). Moreover, pathological diagnosis requires needle biopsy, which is generally only used to diagnose suspected cases.

Cell-free DNA (cfDNA) refers to nucleic acids detected in body fluids and are thought to arise from two sources: passive release through cell death, and active release by cell secretion. Previous studies have shown that it is difficult to improve the sensitivity and specificity of early screening using only a single molecular feature of cfDNA. Therefore, different analytes are used for early screening of cancer, with liquid biopsy being a popular strategy. Recently, we reported a method based upon cfDNA whole-genome sequencing data including the nucleosome distribution (nucleosome footprinting NF), terminal signature sequence (motif), DNA fragmentation (fragment), and copy number variation (CNV) [3]. Here, we propose to implement the cell-free DNA testing method in the patients with ovarian cancer tumors. In addition, we have also assessed an independent series of non-cancer controls to evaluate the specificity of the approach.

## Material and methods

### Patient cohort and epidemiological data acquisition

We collected peripheral blood samples before surgery from a total of 59 patients with OC from Peking Union Medical College Hospital, China, from June 2021 to March 2022. The diagnosis of all patients selected for the experimental group was confirmed cytopathologically and histologically, and the patients had not received surgery, chemoradiotherapy or immunotherapy. In addition, 100 peripheral blood samples were collected from healthy individuals who visited the clinic for routine physical examination. The study was approved by the Ethical Committee of the Peking Union Medical College Hospital, and written informed consent was obtained from all participants according to institutional guidelines.

### Sample processing and cfDNA extraction

All peripheral blood samples were stored in cell-free tubes (Streck, USA) at 4 °C for no more than 72 h before plasma separation by centrifugation at 800 × g for 10 min at 4 °C. The plasma was centrifuged a second time at 18,000 × g at room temperature to remove any remaining cellular debris and stored at − 80 °C until DNA extraction. Plasma cell-free DNA (cfDNA) was isolated using MagMAX Cell-Free DNA Isolation Kit (Thermo, USA) according to the manufacturer's protocol. The quality of the purified DNA was quantified using a Qubit® 4.0 Fluorometer (Life Technologies, USA), and the DNA fragment size composition was assayed with a Fragment Analyzer (Agilent, USA).

### Low-pass whole-genome sequencing and data processing

#### Low-pass WGS library construction and quality inspection

Five nanograms of DNA from each sample was prepared for WGS library construction. The DNA samples were first randomly fragmented and then subjected to end repair/dA tailing (5X ER/A-Tailing Enzyme Mix). dTTP-tailed adapters were ligated to both ends of the repaired/dA-tailed DNA fragments using WGS Ligase and then amplified by PCR. The PCR products from each library were subsequently purified using an Agencourt AMPure XP PCR Purification Kit (Beckman Coulter, Brea, CA, USA); each DNA library was quantified using KAPA Library Quantification Kit (Kapa Biosystems, USA), and sizes were confirmed using a Bioanalyzer (Agilent, USA). The sequencing libraries were pooled at equal amounts and analyzed using the Illumina NovaSeq 6000 platform.

### Processing of low-pass WGS data

FASTQ files were processed with Fastp software (https://github.com/OpenGene/fastp, the detailed parameters are as follows: fastp -i R1.fastq.gz -I R2.fastq.gz –cut_by_quality3 -l 25 –correction -w 8 -o R1.clean.fq.gz -O R2.clean.fq.gz -j jsonfile -h htmlfile) to remove adaptors and sequences with low average sequencing quality together with sequences below 50 bp to acquire clean data. The filtered data were aligned to the Hg19 reference genome using bwa-mem (https://github.com/lh3/bwa, the detailed parameters are as follows: bwa mem-t 4 -M -R '@RG\tID:XJE22A00225_H1\tPL:illumina\tSM:sampleID' hg19.genome.fa R1.clean.fq.gz R2.clean.fq.gz | samtools sort—-m 4G -o sort.bam) to obtain corresponding specific positional information of the genome for each DNA fragment. Data redundancy introduced by PCR was removed using sambamba (https://github.com/biod/sambamba/,the detailed parameters are as follows: sambamba markdup -t 30 sort.bam rmdup.bam –overflow-list-size = 8,000,000 –sort-buffer-size = 10,240) software, and DNA fragments with low alignment quality, unalignment, or not being perfectly paired two-end reads were removed by samtools (http://samtools.sourceforge.net/,the detailed parameters are as follows: samtools view -bh -F 1804 -q 20 rmdup.bam). The filtered DNA fragments were sorted by alignment position for easy subsequent analysis and processing. Reads with mapping rates above 90%, duplicate rates below 25% and coverage above 50% passed the quality control. All sample data were qualified.

### WGS-based biomarker identification and integrated model construction

The characteristic signals unique to cancer patients can be mined by cfDNA WGS data, such as nucleosome footprinting (NF), 5’-end motifs, fragmentation, and CNV. To select more effective biomarkers for distinguishing OC samples from healthy controls, samples were randomly divided into two subsets: the training set consisted of 50 HCs and 40 OC cases, and the test set consisted of the remaining samples (including 50 HCs and 19 OC cases). We constructed a weighted diagnostic model based on the performance of these four features. and the performance of the final model was evaluated using test set data. The detailed selection process was performed as described below.

#### 5’-end motifs

The starting termination position of each DNA fragment was determined by alignment to the reference genome. Then, 256 different types of 4-mer 5’-end motifs were identified, and their percentages were calculated (using pysam (https://pysam.readthedocs.io/en/latest/)) without considering chromosome Y or unidentifiable bases. The following motif types were filtered out: 1) *P* ≥ 0.05 in Wilcoxon rank-sum test between the OC and HC groups; and 2) weight of 0 via LASSO. Eventually, 62 motif types remained for further analysis.

#### **Nucleosome footprinting (NF)**

The main transcripts of coding genes were used for analysis. The promoter region and background region of transcripts were divided, and the read number of different regions was counted with featureCounts [4]. The following genes were filtered out: 1) more than 10% of the total samples showing an NF score of 0; 2) *P* ≥ 0.001 in the Wilcoxon rank-sum test between the ESCC and HC groups; and 3) weight of 0 via LASSO. Eventually, 209 genes remained for further analysis.

#### **Fragments**

The whole genome except for the Y chromosome was divided into 1-M-sized bins, resulting in 3055 areas. Pysam (https://pysam.readthedocs.io/en/latest/) was used to calculate the length of the insertion fragment and the ratio of short/long fragments in different regions. LASSO was then used to filter out areas with a weight of 0; 66 areas were retained.

#### **Copy number variation (CNV)**

The human genome was divided into 2-kb regions, and the average sequencing depth of each was counted and the GC content corrected. A baseline threshold was established for each region with the mean and variance of the copy number from the data for the healthy population. Regions with significant copy number differences were identified compared with the baseline threshold. By connecting them with adjacent windows, CNV regions with a length greater than 2 Mb were obtained. The CNV score was then calculated using an equation reported previously [5].

## OC score model construction

Based on the markers screened by LASSO in the three genomic features, the support vector machine (SVM) method was used for model construction. The combination of parameters in the training set was optimized by means of tenfold cross-validation, and the cutoff value was set at the point with the highest diagnostic accuracy. To obtain the best surveillance model, a logistic regression model was generated based on the prediction score of the single-genomic feature model as input features. The logical score is calculated as follows:

Logistic Score = exp(Z)/(1 + exp(Z)), where Z = -2.48 + (2.84*NF) + (2.01*Fragment) + (0.56*Motif)

Receiver operating characteristic (ROC) curves [6] were generated to evaluate the performance of a prediction algorithm using the pROC [7] library in the R package. Sensitivity and specificity were assessed at the score cutoff that maximizes the sum of sensitivity and specificity using the ROCR library in R.

Finally, the CNV score and logic score were integrated to obtain the OC score.

$$\mathbf{O}\mathbf{C} \mathbf{s}\mathbf{c}\mathbf{o}\mathbf{r}\mathbf{e}=\mathbf{L}\mathbf{o}\mathbf{g}\mathbf{i}\mathbf{s}\mathbf{t}\mathbf{i}\mathbf{c} \mathbf{S}\mathbf{c}\mathbf{o}\mathbf{r}\mathbf{e}$$+CNV Score

## Results

### Sample composition and study design

**Fig. 1:**
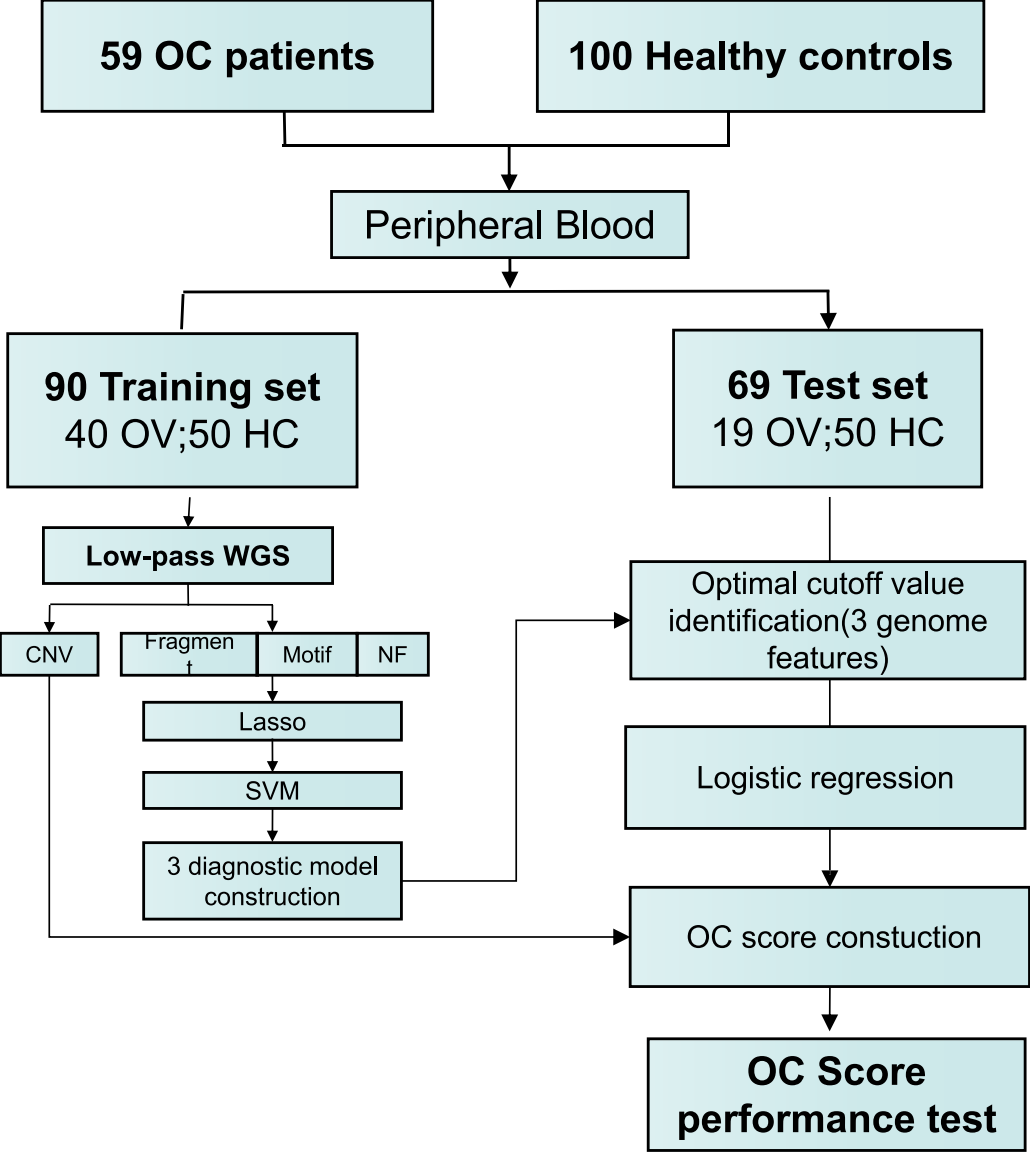
Study design for detection of OC. A diagnostic model based on low-pass WGS was used to identify ctDNA from plasma cfDNA using machine learning methods. 159 participants were randomly split into a training cohort (N = 90) and test cohort (N = 69).OC,ovarian cancer; HC, healthy control; WGS, whole genome sequencing; NF, Nucleosome Footprint; Lasso, Least absolute shrinkage and selection operator; SVM, Support Vector Machine

A total of 59 primary OC patients were retrospectively enrolled in this study, including 10 at FIGO stage I, 10 at stage II, 31 at stage III, and 8 at stage IV. The histologic types of the OCs were high grade serous ovarian cancer (*n* = 38), endometrioid ovarian cancer(*n* = 12), clear cell ovarian cancer(*n* = 4), mucinous ovarian cancer (*n* = 2), brenner ovarian cancer(*n* = 1), undifferentiated ovarian cancer(*n* = 1), and mixed ovarian cancer (serous and sarcoma, *n* = 1). The HC cohort was assembled based on the study criterion of no form of cancer. Detailed pathological and clinical information was obtained from clinical records, as provided in Table 1 and supplementary Table S1. Based on the Wilcoxon test (*P* value = 0.53), the age distribution among the HC and OC groups was unbiased. Our primary aim was to develop a convenient and excellent diagnostic model that reflects the genome-wide features of plasma cfDNA using liquid biopsy techniques to distinguish OC patients from HCs. To select suitable biomarkers and build classification models, OC patients and HCs were randomly divided into two groups: approximately 2/3 of the OC patients were used as the training set (including 50 HCs and 40 OC cases) and 1/3 were used as the test set (including 50 HCs and 19 OC cases) (Fig. 1).

**Table 1:** Summary of demographic and clinicopathological characteristics of all the participants in this study

**Characteristics**		**Healthy controls(*n* = 100)**	**OC patients (*n* = 59)**
**Demographic**			
** Age at surgery, years, n(%)**	< 60	68(68.0)	36(61.0)
	≧60	32(32.0)	23(39.0)
** Gender, n (%)**	Famale	100(100.0)	59(100.0)
**Clinical**			
** FIGO stage, n (%)**	I	-	10(16.9)
	II	-	10(16.9)
	III	-	31(52.6)
	IV	-	8(13.6)
** WHO Classification,n(%)**	Serous	-	38(64.4)
	Endometrioid	-	12(20.3)
	Clear cell	-	4(6.8)
	Mucinous	-	2(3.4)
	Brenner	-	1(1.7)
	Undifferentiated	-	1(1.7)
	Mixed	-	1(1.7)

*Note: OC* ovarian cancer, *WHO* World Health Organization

### Genomic alteration features of ovarian cancer

We analyzed each plasma DNA fragment using massively parallel sequencing. Four genomic feature-based fragments were profiled to determine their relevance in distinguishing OC patients from HCs (Fig. 1).

#### **Fragment size**

**Fig. 2:**
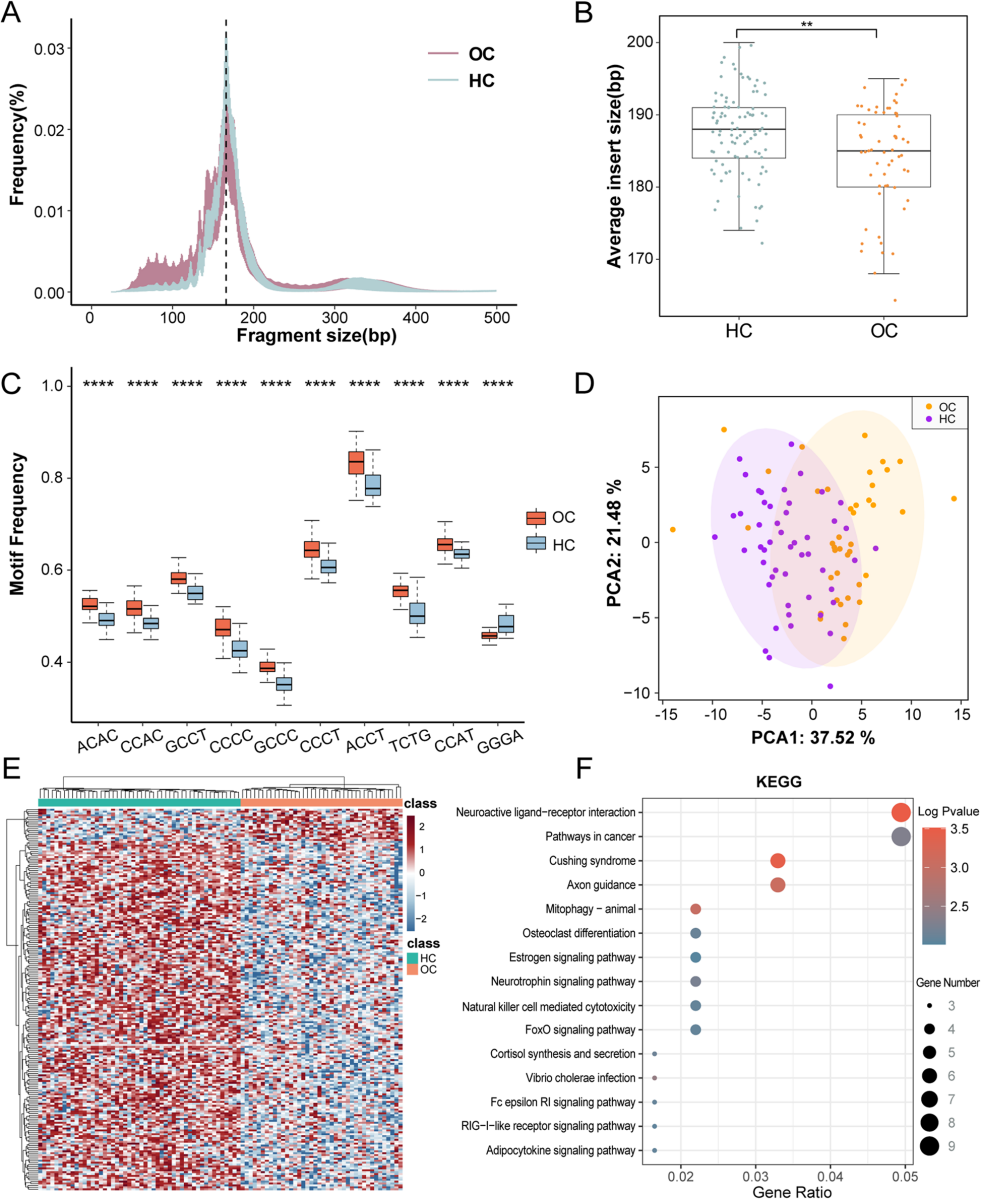
Model construction of genomic features. **A **Fragment size distributions of OC and HC individuals . A reproducible peak in fragment length at 167 bp (black dashed line) is consistent with association with chromatosomes. **B **Box plots showing average insert size of healthy individuals (n = 50) is longer than that of patients with ovarian cancer (n=40). **C** Box plot analysis of Top ten representative motifs showing differential frequencies between OC and HC subjects. **D **Principal Component Analysis(PCA) of training set(including 50HC,40 OC). **E** Heat map classification using 209 genes with different nucleosome distribution. **F **KEGG analysis of 209 genes

We focused on the fragmentation size of cfDNA, as previous studies have shown that the length of cfDNA from cancer cells may be more variable than that from noncancer cells [8]. The size distributions of cfDNA showed that fragment sizes in tumor patients were shorter than those of cfDNA in healthy people (Fig. 2A), and the mean difference in average insert size between the two groups was 3.5 bp (95% confidence interval (CI), 1.3–5.7 bp) (Fig. 2B). To assess differences in fragment size and coverage in a position-dependent manner throughout the genome, the fragments were mapped to the genome, and the whole genome except for the Y chromosome was divided into 1-M-sized bins, resulting in 3055 areas. We calculated the length and coverage of the insertion fragment and the ratio of short (90–150 bp)/long (151–220 bp) fragments in different regions. The genome-wide fragmentation profiles of the training set (50 HCs, 40 OC cases) are shown in Supplementary Fig. 1A. For healthy people, the ratio of short fragments to long fragments was relatively stable and concentrated. In contrast, in OC patients, ratios were more variable. An increase in the proportion indicates an increase in short cfDNA, which is consistent with the characteristics of ctDNA fragmentation in tumor tissues [9]. Differential analysis of the genomic windows of the OC and HC groups revealed a total of 66 windows exhibiting extremely significant proportional differences in short-long fragments, highlighting position-dependent alterations of cfDNA fragments, and the features of these 66 windows can be used to distinguish OC patients from healthy populations.

#### **5’-end motifs**

DNA fragments are nonrandomly cut into cfDNA by the nuclease, and each endonuclease has a preferred base type [10, 11]. It has been demonstrated that the first 4-nucleotide (i.e., 4-mer) sequence of each 5' fragment end of plasma DNA, as the specifically selected break end, carries information regarding the tissue-origin profile of the cfDNA [12]. cfDNA end motifs (4-mer) were identified after alignment to the reference genome (see Methods). By counting the frequency of each plasma DNA end motif, we found a difference in the proportion of base combination types at the ends of fragments from a genome-wide perspective. Hierarchical clustering analysis of the 256 motif frequencies revealed that OC samples tended to cluster together but that HC samples tended to form different clusters (Supplementary Fig. 1B). By P value filtering (Wilcoxon test, *P* value < 0.05) and LASSO dimension reduction,62 motifs significantly different between the OC and HC groups were selected. The motif features of top 10 in the training set are listed in the boxplot, and these motifs between the OC and HC groups exhibited significant differences. Among the top 10 motifs, most showed a significant increase in patients with OC (e.g., ACAA, CCAC), though GGGA was significantly decreased in OC patients (Fig. 2C). PCA demonstrated the ability of 62 motifs to act as a potential classification parameter (Fig. 2D).

#### **Nucleosome footprinting (NF)**

Nucleosomes protect the DNA structure from endogenous nuclease activity. In regions with low transcriptional activity, nucleosomes are closely arranged; however, the chromosome structure is usually loose in malignant tumors, and there are few nucleosomes in regions with active gene transcription and expression [13]. Hence, we determined the coverage of nucleosome degraded (NDR, from − 150 BP to + 50 BP with respect to the TSS) and the background (from − 2000 BP to + 2000 BP with respect to the TSS) regions of all coding genes. The difference in coverage of the background region and NDR represents the dispersion of nucleosome distribution. After screening, we obtained 209 genes with different nucleosome distributions in patients with OC and in healthy controls. NF heatmap analysis indicated that genes with differential read coverage between promoter and background regions are able to distinguish OC patients from HCs (Fig. 2E). Moreover, KEGG enrichment results showed the differentially expressed genes to be enriched in neuroactive ligand − receptor interactions, pathways in cancer and estrogen signaling pathways (Fig. 2F).

We found CNV signals in 17/59 OC samples, and the CNVs are provided in Supplementary Table S2. Collectively, all four genome features of cfDNA showed promising diagnostic potential for OC.

### Performance evaluation of the OC score for individuals with OC

**Fig. 3:**
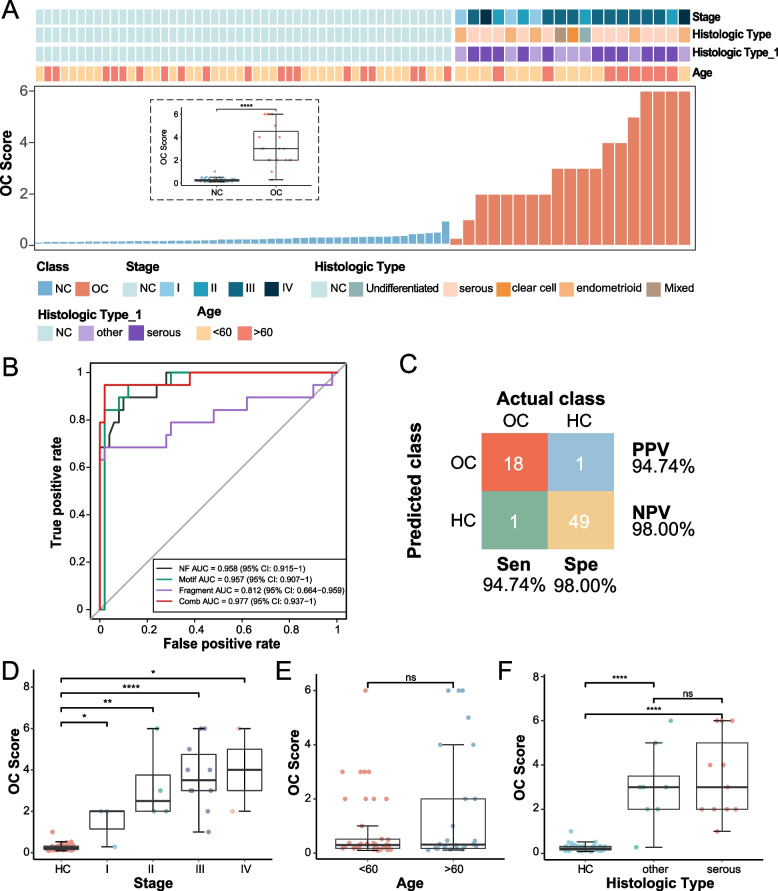
Diagnostic value of OC score in ovarian cancer. **A **The OC scores of all participants in the test cohort. Upper: clinical characteristics of all sample. **B **ROC curves of OC score and solitary genomic features for OC patients versus HC individuals in the test cohort. **C** Confusion matrices showing OC score for diagnosis performance in the test cohort. **D **The sensitivity performance of OC score in different stage at specificity of 98.0%. **E **The sensitivity performance of OC score in different age group. **F **The sensitivity performance of OC score in different histologic type

By integrating the above four-dimensional genomic features, a multidimensional OC early warning model was constructed. This model was used to assess the genomic features of each subject in the validation cohort and provide an independent score (OC score). OC patients and HCs showed extremely significant differences in scores (Fig. 3A). The OC score also exhibited strongly sensitive detection and diagnostic potential in different clinical subgroups, particularly for histological subtypes and FIGO staging, but not in different age groups (Fig. 3D-F); it showed a strong diagnostic value in distinguishing between the OC and HC groups. we grouped according to the current metastatic status and resectability of the tumors, and a one-way ANOVA was conducted to compare OC score in the different groups (Supplementary Fig. 2A-B). OC score was significantly different between the metastatic and non-metastatic groups(mean ± SD: 3.73 ± 1.71; 1.42 ± 0.99; respectively; one-way ANOVA, F = 4.96, *P* < 0.05). OC score was significantly different between the resectable and non-resectable groups(mean ± SD: 1.57 ± 0.86; 3.86 ± 1.70; respectively; one-way ANOVA, F = 6.52, *P* < 0.05). The sensitivity and specificity of the model were 94.74% and 98.00%, respectively, in the independent test set (19 OC cases and 50 HCs) (Fig. 3C). In the test set, the area under the curve (AUC) for NF, motif, and fragment was 0.958, 0.957, and 0.812, respectively; that of the OC score was 0.977 (Fig. 3B). Motif had the highest sensitivity (94.7%) and fragment the highest specificity (98%). Detection of cfDNA by CNV exhibited good performance with regard to PPV (100%) and specificity (100%), though the sensitivity was poor (36.84%). These parameters showed both advantages and disadvantages. The accuracy of the three genomic features (NF, motif and fragment) was 89.86%; that for CNV was 82.61%. Thus, OC diagnostic models based on multiple genomic features can effectively distinguish OC patients from HCs (AUC = 0.977[0.937–1.000]), and their diagnostic performance is significantly higher than that of any diagnostic model built using a single genomic feature.

## Discussion

The development of gene sequencing technology has allowed for continued maturation of cfDNA detection technology, and its clinical application potential in tumor diagnosis and treatment is very wide. In addition to detecting somatic mutations in plasma to obtain cancer occurrence signals, abnormalities can also be found in terminal characteristic sequences (motif), nucleosome distribution (NF), DNA fragmentation (fragment), and CNVs, among others.

This study is the first to use genome-wide multidimensional variation indicators, including terminal characteristic sequences (motif), nucleosome distribution (NF), DNA fragmentation (fragment), and copy number abnormalities, to construct a multidimensional whole-genome model for OC. We developed a genetic screening model to distinguish patients with OC from HCs and demonstrated the advantages of this multidimensional genomics diagnostic model over the traditional biomarker CA125.

Based on the unique genomic features of cfDNA, we developed a new comprehensive OC diagnosis method, the OC score, which has high accuracy in distinguishing OC patients from HCs (AUC 97.7%; sensitivity 94.7%; specificity 98.0%), with good application in clinical practice. Compared with imaging and pathology detection, serology has the characteristics of noninvasiveness, rapidity, ability to detect trace amounts and high patient compliance. However, traditional serological markers have certain limitations, such as the insufficient sensitivity and specificity of CA125 and elevated concentrations present in some benign diseases; furthermore, low levels of markers cannot rule out the possibility of OC. HE4 has high specificity, but its concentration also increases in other malignant tumors, with age and renal function also having certain effects. For these reasons, in an attempt to improve the inherent characteristics of these biomarkers, some studies have integrated CA125 with HE4 and developed the ROMA algorithm. Overall, the ROMA algorithm is more sensitive but less specific than HE4 alone [14]. Detection of cfDNA can capture free tumor DNA fragments (ctDNA) in the blood and mine the unique characteristic signals of tumor patients.

As ctDNA is derived from the tumor genome and carries relevant information about the tumor, it has a wide range of potential clinical applications, mainly involving early tumor screening, tumor dynamic monitoring, and recurrence risk. At present, there are two main problems hindering its development: the low abundance of ctDNA and the background cfDNA noise in the blood, which affect the sensitivity and reliability of the diagnosis. Whole-genome sequencing (WGS) overcomes these problems through advantages of high coverage, high throughput and high resolution. Several studies have elucidated the molecular features of ctDNA. Nucleosomes are the basic structural units of chromatin formed by DNA and histones that protect DNA structures from damage due to endogenous nuclease activity. In an organism, DNA in each cell type is packed slightly differently and in different functional regions of the genome, and these differences leave indicator marks in the resulting cfDNA. Therefore, the distribution of nucleosomes has a certain tumor specificity [13, 15]. Different exonucleases degrade genomic DNA to form cfDNA with specific end motifs. In general, the diversity of DNA end sequences in the plasma of cancer patients is significantly increased, and it is highly preferred. Healthy cfDNA mainly derives from programmed or natural cell death; the proportion of ctDNA in cfDNA increases in disease, and its fragment size is shorter than that in healthy people [8]. In addition, cancer patients often have chromosomal abnormalities such as CNVs, and CNV signals in tumor tissue can be stably detected at an average depth of 1 × genome coverage [16, 17]. As a consequence, specific genomic signatures of cfDNA detected by WGS can provide precise information about tumor cell populations. Methylation detection of ctDNA in peripheral blood is a popular non-invasive early diagnosis method in the field of tumor detection in recent years, and it is also common in the field of ovarian cancer. Biomarkers based on tumor specific methylation have proved to be of great value in monitoring the prognosis of diseases and different pathological determinants [18]. As mentioned above, many studies have reported abnormal methylation in ovarian cancer [19, 20], and also used methylation as a classification feature to verify its role in diagnosis. However, compared with the single dimension methylation sequencing technology, this study uses multi-dimensional variation indicators within the whole genome, with stable performance, wider coverage and greater overall accuracy. At the same time, the ability of whole genome sequencing technology to expand application scenarios is relatively strong. This article does not make detailed research on each subspecies, such as the determination of benign and malignant diseases. In this article, we did not conduct detailed research on each subspecies, such as the judgment of benign and malignant, etc. If study want to distinguish between subspecies and benign and malignant ovarian diseases in the future, there is no need to switch the experimental process. It is enough to replace the indicator in the process, but methylation needs to redesign the probe to find the target. At the same time, the low-pass whole-genome sequencing used in this study is relatively cheap, and more suitable for large-scale expansion in the field of early screening of ovarian cancer.

Above all, ctDNA can be used as a potential biomarker for the diagnosis of OC. As previous studies have shown that it is difficult to improve the sensitivity and specificity of early screening only by a single molecular feature in ctDNA, the combination of different analytes has been used for early cancer screening, and liquid biopsy is an inevitable trend. Based on the performance found in this study, the sensitivity and specificity of NF were similar (AUC 95.8%; sensitivity 89.5%; specificity 90.0%), whereas the sensitivity of motif was excellent but specificity slightly insufficient (AUC 95.7%; sensitivity 94.7%; specificity 88.0%); the fragment distribution trended in the opposite direction (AUC 81.2%; sensitivity 68.4%; specificity 98.0%%). Conversely, CNV showed excellent specificity but low sensitivity (sensitivity 36.8%; specificity 100.0%). The OC score, which integrates multiple features, had a sensitivity and specificity of 94.74% and 98.00%, respectively, with obvious advantages over traditional serum markers and single-omics indicators. In assessing the diagnostic value of OC score in a subgroup of ovarian cancer patients, OC score accurately distinguishes between CA125-negative ovarian cancer patients (Supplementary Fig. 2C) We maintained a 100% detection rate in ca125-positive patients, and we still had a 60% detection rate in ca125-negative patients. And because the sample size of this study was insufficient, this proportion might have been higher if in the real world.

Although early detection of OC reduces mortality by 10–30%, unfortunately, only 15% of OCs are diagnosed at an early or localized stage [21]. Therefore, early screening of OC has always been a clinical concern. In this study, the detection rate of early OC reached 85.7% (I, II), which is promising for early screening of high-risk groups. Regardless, the performance of the model needs to be validated in a larger cohort.

The present results demonstrate the great value of ctDNA-based fragmentomic analysis in OC diagnosis as a noninvasive tool with potential to improve early cancer screening modalities at an acceptable cost in routine clinical settings, with specificity and sensitivity. In the routine clinical environment, cancer screening can be carried out with high specificity and sensitivity at an acceptable cost to significantly reduce cancer-related mortality. Nonetheless, further studies with larger cohorts are needed to determine which ctDNA signatures are most accurate when applied to large populations of patients and should include more early-stage samples to develop models that are more suitable for early-stage patients.

## Supplementary Information

**Additional file 1: Table S1. **Clinicopathological characteristics of ovarian cancer patients and healthy controls. **Table S2. **Copy number variation in 17 ovarian cancer samples

**Additional file 2: Supplementary Fig. 1.** Genome-wide cfDNA fragmentation and end motif profiles. (A) Genome-wide cfDNA fragmentation profiles (defined as the ratio of short to long fragments) are shown in 1-Mb bins for 50 healthy individuals (top) and 40 patients with ovarian cancer (bottom). (B) Heat map classification using 256 motif.

**Additional file 3: Supplementary Fig. 2.** The detection ability of OC score in different clinical subgroups. (A-B) The ability of OC score to distinguish resectability and metastatic status. (C) Detection rate of OC score in different CA125 contents.

## Data Availability

All data generated or analysed during this study are included in the published article.

## Abbreviations

cfDNA: Cell-free DNA
OC: Ovarian cancer
HC: Healthy control
CNV: Copy number variation
NF: Nucleosome footprinting
WGS: Whole-genome sequencing
ROC: Receiver operating characteristic
SVM: Support vector machine
FIGO: International Federation of Obstetrics and Gynecology
