# Supplementary material for: Can circulating cell free DNA be a promising marker in ovarian cancer? – a genome-scale profiling study in a single institution
Source: J Ovarian Res. 2023 Jan 14;16:11. doi: 10.1186/s13048-022-01068-z (PMC9840311; doi:10.1186/s13048-022-01068-z)
Supplement: Supplementary file 1 — Additional file 1: Table S1. Clinicopathological characteristics of ovarian cancer patients and healthy controls. Table S2. Copy number variation in 17 ovarian cancer samples [file 13048_2022_1068_MOESM1_ESM.docx]

| **Table S1 Clinicopathological characteristics of ovarian cancer patients and healthy controls.** | | | | | | |  |
| --- | --- | --- | --- | --- | --- | --- | --- |
| **Sample ID** | **Population** | **Group** | **Age** | **histologic_type** | **FIGO stage** | **CNV numer** | **score** |
| ZL22N0039 | Chinese | Train | 53 | endometrioid | II | 0 | 2 |
| ZL22N0040 | Chinese | Train | 47 | serous | IIIC | 0 | 2 |
| ZL22N0001 | Chinese | Train | 27 | clearcell | IIB | 0 | 2 |
| Z21S03128 | Chinese | Train | 53 | serous | IIIC | 88 | 6 |
| ZL22N0029 | Chinese | Test | 50 | endometrioid | IA | 0 | 0.28247825 |
| Z20S00751 | Chinese | Train | 58 | mucinous | IIIC | 0 | 3 |
| ZL22N0035 | Chinese | Train | 54 | serous | IIIC | 95 | 6 |
| Z21S02186 | Chinese | Test | 41 | serous | IIIC | 0 | 1 |
| Z21S02539 | Chinese | Test | 56 | serous | IVB | 0 | 2 |
| Z21S02541 | Chinese | Train | 52 | serous | IIIC | 0 | 3 |
| Z21S02537 | Chinese | Test | 82 | serous | IIB | 0 | 2 |
| Z21S02536 | Chinese | Train | 79 | serous | IIIC | 0 | 3 |
| Z21S02538 | Chinese | Test | 38 | endometrioid | IC | 0 | 2 |
| Z21S02535 | Chinese | Train | 55 | clearcell | IIIC | 0 | 3 |
| Z21S02545 | Chinese | Test | 52 | serous | IIB | 0 | 2 |
| ZL22N0036 | Chinese | Train | 66 | serous | IIIC | 67 | 6 |
| Z21S00770 | Chinese | Test | 37 | endometrioid | IC2 | 0 | 2 |
| Z21S02549 | Chinese | Train | 50 | endometrioid | IIIB | 0 | 3 |
| Z21S02550 | Chinese | Train | 34 | serous | IIB | 1 | 2 |
| Z21S03125 | Chinese | Test | 78 | serous | IIIB | 0 | 2 |
| Z20S00752 | Chinese | Train | 63 | serous | IIIB | 7 | 6 |
| Z21S00784 | Chinese | Train | 36 | serous | IIIA | 0 | 3 |
| Z21S00786 | Chinese | Train | 69 | endometrioid | IA | 0 | 3 |
| Z21S00787 | Chinese | Train | 73 | brenner | I | 0 | 3 |
| Z21S00783 | Chinese | Train | 66 | serous | IIB | 0 | 3 |
| Z21S00791 | Chinese | Train | 67 | serous | IIIC | 38 | 6 |
| Z21S02546 | Chinese | Train | 65 | serous | IVB | 0 | 2 |
| Z21S02543 | Chinese | Test | 56 | Mixed | IIIC | 0 | 3 |
| Z21S03129 | Chinese | Test | 55 | clearcell | IIIC | 0 | 3 |
| Z21S03170 | Chinese | Train | 55 | serous | IVB | 0 | 3 |
| Z21S03127 | Chinese | Train | 29 | mucinous | IA | 0 | 2 |
| Z21S03528 | Chinese | Test | 49 | Undifferentiated | IIB | 0 | 3 |
| Z21S03692 | Chinese | Train | 51 | endometrioid | IC | 35 | 6 |
| Z21S03126 | Chinese | Train | 64 | serous | IIIC | 0 | 3 |
| Z21S03700 | Chinese | Train | 61 | serous | IVB | 0 | 3 |
| Z21S03694 | Chinese | Train | 45 | serous | IIA | 0 | 3 |
| ZL22N0037 | Chinese | Test | 50 | serous | IIIC | 0 | 3 |
| Z21S03691 | Chinese | Train | 42 | serous | IIIC | 0 | 3 |
| ZL22N0002 | Chinese | Test | 70 | serous | IIIC | 9 | 4 |
| Z21S03696 | Chinese | Train | 57 | serous | IIIC | 0 | 3 |
| Z21S02548 | Chinese | Train | 78 | endometrioid | IA | 12 | 6 |
| Z21S00777 | Chinese | Train | 70 | serous | IIIC | 16 | 4 |
| Z21S03695 | Chinese | Train | 36 | endometrioid | IC | 0 | 3 |
| Z21S04934 | Chinese | Train | 71 | serous | IIIC | 0 | 2 |
| Z21S03693 | Chinese | Test | 61 | serous | IIIC | 1 | 4 |
| Z21S02272 | Chinese | Train | 44 | serous | IVB | 0 | 3 |
| Z21S03697 | Chinese | Test | 68 | endometrioid | IIIC | 8 | 5 |
| Z21S02544 | Chinese | Test | 67 | serous | IIIC | 69 | 6 |
| ZL22N0032 | Chinese | Train | 72 | serous | IIIC | 2 | 3 |
| ZL22N0033 | Chinese | Train | 73 | serous | IIB | 0 | 3 |
| Z21S02540 | Chinese | Test | 64 | serous | IIIC | 25 | 6 |
| Z21S02269 | Chinese | Train | 56 | clearcell | IVB | 2 | 4 |
| Z21S03698 | Chinese | Train | 58 | serous | IIIC | 4 | 4 |
| ZL22N0030 | Chinese | Train | 59 | serous | IVA | 0 | 2 |
| Z21S02532 | Chinese | Test | 74 | serous | IIB | 11 | 6 |
| ZL22N0034 | Chinese | Train | 39 | endometrioid | IC | 0 | 2 |
| Z21S00757 | Chinese | Train | 57 | serous | IIIC | 0 | 2 |
| ZL22N0031 | Chinese | Test | 55 | endometrioid | IVB | 6 | 6 |
| Z21S02542 | Chinese | Train | 53 | serous | IIIB | 0 | 2 |
| Y19KB033CV322 | Chinese | Train | 52 | NC | NC | 0 | 0.10132568 |
| Y19KB048CV388 | Chinese | Train | 63 | NC | NC | 0 | 0.14091396 |
| Y19KB052CV362 | Chinese | Train | 59 | NC | NC | 0 | 0.10741904 |
| Y19KB075cV337 | Chinese | Train | 56 | NC | NC | 0 | 0.12540196 |
| Y19KB077CV338 | Chinese | Train | 63 | NC | NC | 0 | 0.1160696 |
| Y19KB079CV351 | Chinese | Train | 49 | NC | NC | 0 | 0.13859827 |
| Y19KB089CV386 | Chinese | Train | 51 | NC | NC | 0 | 0.0930793 |
| Y19KB097CV383 | Chinese | Train | 56 | NC | NC | 0 | 0.10083116 |
| Y19KB099CV381 | Chinese | Train | 58 | NC | NC | 0 | 0.11680223 |
| Y19KB103CV368 | Chinese | Train | 64 | NC | NC | 0 | 0.15587807 |
| Y19KB157V326 | Chinese | Train | 49 | NC | NC | 0 | 0.10080678 |
| Y19KB163V330 | Chinese | Train | 67 | NC | NC | 0 | 0.09885538 |
| Y19KB179V332 | Chinese | Train | 55 | NC | NC | 0 | 0.10498722 |
| Y19KB320V380 | Chinese | Train | 54 | NC | NC | 0 | 0.10850741 |
| Y19KB323V381 | Chinese | Train | 52 | NC | NC | 0 | 0.10028624 |
| Y19KB400V336 | Chinese | Train | 43 | NC | NC | 0 | 0.14191281 |
| Y21KC001CV315 | Chinese | Test | 59 | NC | NC | 0 | 0.34233411 |
| Y21KC003C-317A | Chinese | Test | 58 | NC | NC | 0 | 0.13070246 |
| Y21KC004C-325A | Chinese | Test | 62 | NC | NC | 0 | 0.28742872 |
| Y21KC010C-373A | Chinese | Test | 66 | NC | NC | 0 | 0.14298509 |
| Y21KC013CV377 | Chinese | Test | 43 | NC | NC | 0 | 0.34868141 |
| Y21KC015CV313 | Chinese | Test | 71 | NC | NC | 0 | 0.26559481 |
| Y21KC016CV334 | Chinese | Train | 42 | NC | NC | 0 | 0.12650853 |
| Y21KC017C-335A | Chinese | Test | 47 | NC | NC | 0 | 0.2244446 |
| Y21KC018C-343A | Chinese | Test | 60 | NC | NC | 0 | 0.25999694 |
| Y21KC020C-359A | Chinese | Train | 44 | NC | NC | 0 | 0.1033599 |
| Y21KC021C-367A | Chinese | Train | 40 | NC | NC | 0 | 0.15379594 |
| Y21KC023C-383A | Chinese | Train | 55 | NC | NC | 0 | 0.10783906 |
| Y21KC027C-371A | Chinese | Train | 50 | NC | NC | 0 | 0.09498456 |
| Y21KC030C-395A | Chinese | Train | 52 | NC | NC | 0 | 0.09228027 |
| Y21KC033C-324A | Chinese | Test | 59 | NC | NC | 0 | 0.12386788 |
| Y21KC035C-340A | Chinese | Test | 60 | NC | NC | 0 | 0.33742537 |
| Y21KC036C-348A | Chinese | Train | 61 | NC | NC | 0 | 0.0901318 |
| Y21KC038C-364A | Chinese | Test | 61 | NC | NC | 0 | 0.1834811 |
| Y21KC045C-325A | Chinese | Test | 66 | NC | NC | 0 | 0.22890129 |
| Y21KC046C-333A | Chinese | Train | 63 | NC | NC | 0 | 0.10462773 |
| Y21KC049CV353 | Chinese | Test | 57 | NC | NC | 0 | 0.36741532 |
| Y21KC051C-373A | Chinese | Train | 47 | NC | NC | 0 | 0.0895253 |
| Y21KC056C-319A | Chinese | Test | 53 | NC | NC | 0 | 0.31201146 |
| Y21KC057C-327A | Chinese | Train | 62 | NC | NC | 0 | 0.12759558 |
| Y21KC058C-335A | Chinese | Test | 56 | NC | NC | 0 | 0.13693743 |
| Y21KC060C-351A | Chinese | Test | 55 | NC | NC | 0 | 0.52248162 |
| Y21KC062C-367A | Chinese | Train | 57 | NC | NC | 0 | 0.0923094 |
| Y21KC067C-321A | Chinese | Test | 53 | NC | NC | 0 | 0.30496138 |
| Y21KC070C-345A | Chinese | Train | 58 | NC | NC | 0 | 0.09261731 |
| Y21KC073C-369A | Chinese | Test | 67 | NC | NC | 0 | 0.45155674 |
| Y21KC075C-385A | Chinese | Test | 73 | NC | NC | 0 | 0.31529981 |
| Y21KC077C-363A | Chinese | Train | 51 | NC | NC | 0 | 0.20475496 |
| Y21KC078C-371A | Chinese | Test | 57 | NC | NC | 0 | 0.49566978 |
| Y21KC083C-316A | Chinese | Test | 63 | NC | NC | 0 | 0.11607568 |
| Y21KC087C-340A | Chinese | Train | 32 | NC | NC | 0 | 0.08926804 |
| Y21KC088CV360 | Chinese | Train | 45 | NC | NC | 0 | 0.10350093 |
| Y21KC089C-356A | Chinese | Test | 60 | NC | NC | 0 | 0.11395844 |
| Y21KC093CV361 | Chinese | Train | 64 | NC | NC | 0 | 0.13220901 |
| Y21KC094CV391 | Chinese | Test | 59 | NC | NC | 0 | 0.12073214 |
| Y21KC095C-301A | Chinese | Train | 59 | NC | NC | 0 | 0.09233266 |
| Y21KC097C-317A | Chinese | Test | 63 | NC | NC | 0 | 0.17104372 |
| Y21KC099C-325A | Chinese | Test | 59 | NC | NC | 0 | 0.16544523 |
| Y21KC101C-349A | Chinese | Train | 53 | NC | NC | 0 | 0.09589498 |
| Y21KC103C-365A | Chinese | Test | 50 | NC | NC | 0 | 0.11816733 |
| Y21KC104CV389 | Chinese | Test | 45 | NC | NC | 0 | 0.3261064 |
| Y21KC105C-381A | Chinese | Test | 46 | NC | NC | 0 | 0.16340202 |
| Y21KC109C-318A | Chinese | Train | 52 | NC | NC | 0 | 0.09427162 |
| Y21KC112C-342A | Chinese | Test | 65 | NC | NC | 0 | 0.33188341 |
| Y21KC114C-358A | Chinese | Test | 55 | NC | NC | 0 | 0.1766518 |
| Y21KC115C-366A | Chinese | Test | 65 | NC | NC | 0 | 1 |
| Y21KC116C-374A | Chinese | Train | 44 | NC | NC | 0 | 0.09245482 |
| Y21KC118C-390A | Chinese | Test | 53 | NC | NC | 0 | 0.1786526 |
| Y21KC119C-303A | Chinese | Test | 60 | NC | NC | 0 | 0.46785665 |
| Y21KC124C-343A | Chinese | Train | 57 | NC | NC | 0 | 0.09502354 |
| Y21KC126C-359A | Chinese | Train | 52 | NC | NC | 0 | 0.09371264 |
| Y21KC127C-367A | Chinese | Test | 52 | NC | NC | 0 | 0.37263221 |
| Y21KC128CV388 | Chinese | Test | 56 | NC | NC | 0 | 0.30781595 |
| Y21KC130C-391A | Chinese | Test | 47 | NC | NC | 0 | 0.15205424 |
| Y21KC132CV362 | Chinese | Train | 62 | NC | NC | 0 | 0.09464142 |
| Y21KC133C-320A | Chinese | Train | 61 | NC | NC | 0 | 0.09555358 |
| Y21KC135C-336A | Chinese | Train | 59 | NC | NC | 0 | 0.08415978 |
| Y21KC136C-344A | Chinese | Train | 43 | NC | NC | 0 | 0.09849869 |
| Y21KC137C-352A | Chinese | Train | 64 | NC | NC | 0 | 0.0926397 |
| Y21KC139CV359 | Chinese | Test | 55 | NC | NC | 0 | 0.30423103 |
| Y21KC140C-376A | Chinese | Test | 61 | NC | NC | 0 | 0.16385124 |
| Y21KC146C-329A | Chinese | Test | 69 | NC | NC | 0 | 0.16061116 |
| Y21KC148C-345A | Chinese | Test | 58 | NC | NC | 0 | 0.16146398 |
| Y21KC149C-353A | Chinese | Test | 59 | NC | NC | 0 | 0.23734291 |
| Y21KC150C-361A | Chinese | Test | 57 | NC | NC | 0 | 0.0938734 |
| Y21KC152C-377A | Chinese | Train | 65 | NC | NC | 0 | 0.09529211 |
| Y21KC153C-385A | Chinese | Test | 55 | NC | NC | 0 | 0.21876436 |
| Y21KC156C-314A | Chinese | Test | 55 | NC | NC | 0 | 0.25423496 |
| Y21KC157C-322A | Chinese | Test | 44 | NC | NC | 0 | 0.19439455 |
| Y21KC159C-338A | Chinese | Train | 42 | NC | NC | 0 | 0.09730976 |
| Y21KC161C-370A | Chinese | Train | 44 | NC | NC | 0 | 0.09295482 |
| Y21KC162C-378A | Chinese | Test | 54 | NC | NC | 0 | 0.23375089 |
| Y21KC167C-323A | Chinese | Train | 58 | NC | NC | 0 | 0.14253463 |
| Y21KC169CV393 | Chinese | Train | 50 | NC | NC | 0 | 0.0953869 |
| Y21KC171C-355A | Chinese | Train | 46 | NC | NC | 0 | 0.10606091 |
| Y21KC172C-363A | Chinese | Test | 66 | NC | NC | 0 | 0.1447371 |
| Y21KC173CV348 | Chinese | Test | 40 | NC | NC | 0 | 0.29595594 |
| Y21KC176C-387A | Chinese | Test | 65 | NC | NC | 0 | 0.13790289 |
| Y21KC181CV345 | Chinese | Train | 63 | NC | NC | 0 | 0.11404077 |
| Y21KC194CV394 | Chinese | Test | 50 | NC | NC | 0 | 0.22751048 |

| **Table S2 Copy number variation in 17 ovarian cancer samples** | | | | | |
| --- | --- | --- | --- | --- | --- |
| **Sample** | **Chr** | **Start** | **End** | **CopyNum** | **class** |
| Z21S00777 | chr1 | 800000 | 34100000 | 1.936 | del |
| Z21S00777 | chr1 | 144800000 | 249200000 | 2.134 | dup |
| Z21S00777 | chr3 | 100000 | 90500000 | 2.069 | dup |
| Z21S00777 | chr3 | 93500000 | 197900000 | 2.087 | dup |
| Z21S00777 | chr4 | 52700000 | 190900000 | 1.959 | del |
| Z21S00777 | chr6 | 100000 | 55000000 | 2.221 | dup |
| Z21S00777 | chr6 | 61900000 | 92300000 | 2.044 | dup |
| Z21S00777 | chr6 | 92300000 | 170900000 | 1.957 | del |
| Z21S00777 | chr8 | 46900000 | 146300000 | 2.058 | dup |
| Z21S00777 | chr9 | 200000 | 38800000 | 2.046 | dup |
| Z21S00777 | chr9 | 71000000 | 141100000 | 1.968 | del |
| Z21S00777 | chr11 | 54800000 | 135000000 | 2.039 | dup |
| Z21S00777 | chr14 | 20200000 | 107300000 | 1.965 | del |
| Z21S00777 | chr20 | 0 | 26300000 | 2.058 | dup |
| Z21S00777 | chr20 | 29800000 | 62900000 | 2.069 | dup |
| Z21S00777 | chr21 | 15400000 | 48100000 | 2.033 | dup |
| Z20S00752 | chr4 | 0 | 49100000 | 2.054 | dup |
| Z20S00752 | chr7 | 77400000 | 79600000 | 2.436 | dup |
| Z20S00752 | chr9 | 200000 | 38800000 | 1.968 | del |
| Z20S00752 | chr11 | 109600000 | 135000000 | 1.937 | del |
| Z20S00752 | chr13 | 19400000 | 115100000 | 2.037 | dup |
| Z20S00752 | chr19 | 32800000 | 41000000 | 2.254 | dup |
| Z20S00752 | chr21 | 15400000 | 48100000 | 1.964 | del |
| Z21S02540 | chr1 | 5000000 | 37100000 | 1.959 | del |
| Z21S02540 | chr1 | 37700000 | 68600000 | 1.941 | del |
| Z21S02540 | chr1 | 177500000 | 234600000 | 1.951 | del |
| Z21S02540 | chr3 | 93500000 | 114100000 | 2.046 | dup |
| Z21S02540 | chr3 | 114900000 | 186300000 | 2.067 | dup |
| Z21S02540 | chr3 | 186300000 | 197900000 | 1.905 | del |
| Z21S02540 | chr5 | 0 | 34600000 | 2.179 | dup |
| Z21S02540 | chr5 | 34600000 | 46400000 | 2.068 | dup |
| Z21S02540 | chr5 | 49500000 | 143400000 | 1.964 | del |
| Z21S02540 | chr5 | 143400000 | 180700000 | 2.061 | dup |
| Z21S02540 | chr6 | 100000 | 10700000 | 2.079 | dup |
| Z21S02540 | chr6 | 61900000 | 164600000 | 1.964 | del |
| Z21S02540 | chr7 | 65900000 | 116800000 | 1.97 | del |
| Z21S02540 | chr7 | 116800000 | 152500000 | 2.094 | dup |
| Z21S02540 | chr7 | 152500000 | 159100000 | 2.237 | dup |
| Z21S02540 | chr8 | 106700000 | 146300000 | 2.16 | dup |
| Z21S02540 | chr9 | 200000 | 38800000 | 2.071 | dup |
| Z21S02540 | chr11 | 54800000 | 87200000 | 1.943 | del |
| Z21S02540 | chr12 | 100000 | 34900000 | 1.94 | del |
| Z21S02540 | chr12 | 38700000 | 125500000 | 1.95 | del |
| Z21S02540 | chr12 | 125500000 | 133851895 | 2.073 | dup |
| Z21S02540 | chr13 | 19400000 | 115100000 | 1.945 | del |
| Z21S02540 | chr15 | 20500000 | 102400000 | 1.931 | del |
| Z21S02540 | chr19 | 27700000 | 42900000 | 2.142 | dup |
| Z21S02540 | chr21 | 15400000 | 48100000 | 1.954 | del |
| Z21S02550 | chr5 | 0 | 46400000 | 1.957 | del |
| Z21S00791 | chr1 | 800000 | 24900000 | 2.048 | dup |
| Z21S00791 | chr1 | 24900000 | 118900000 | 1.94 | del |
| Z21S00791 | chr1 | 144800000 | 154100000 | 2.133 | dup |
| Z21S00791 | chr2 | 0 | 39200000 | 2.154 | dup |
| Z21S00791 | chr2 | 95500000 | 144100000 | 2.041 | dup |
| Z21S00791 | chr2 | 146300000 | 233100000 | 2.048 | dup |
| Z21S00791 | chr2 | 233100000 | 243100000 | 1.92 | del |
| Z21S00791 | chr3 | 9100000 | 80600000 | 1.934 | del |
| Z21S00791 | chr3 | 93500000 | 158500000 | 2.035 | dup |
| Z21S00791 | chr3 | 158500000 | 197900000 | 2.106 | dup |
| Z21S00791 | chr4 | 0 | 49100000 | 2.052 | dup |
| Z21S00791 | chr4 | 52700000 | 73500000 | 2.041 | dup |
| Z21S00791 | chr4 | 73500000 | 190900000 | 1.931 | del |
| Z21S00791 | chr5 | 49500000 | 180700000 | 1.95 | del |
| Z21S00791 | chr6 | 100000 | 22800000 | 2.112 | dup |
| Z21S00791 | chr7 | 0 | 58000000 | 1.955 | del |
| Z21S00791 | chr8 | 100000 | 21500000 | 2.078 | dup |
| Z21S00791 | chr8 | 21500000 | 43800000 | 1.937 | del |
| Z21S00791 | chr8 | 46900000 | 63500000 | 2.072 | dup |
| Z21S00791 | chr8 | 63500000 | 138100000 | 2.203 | dup |
| Z21S00791 | chr8 | 138100000 | 146300000 | 2.08 | dup |
| Z21S00791 | chr9 | 200000 | 31200000 | 1.929 | del |
| Z21S00791 | chr9 | 31200000 | 38800000 | 2.072 | dup |
| Z21S00791 | chr9 | 71000000 | 141100000 | 2.048 | dup |
| Z21S00791 | chr10 | 100000 | 39200000 | 1.928 | del |
| Z21S00791 | chr10 | 42400000 | 74900000 | 2.035 | dup |
| Z21S00791 | chr10 | 74900000 | 118800000 | 1.95 | del |
| Z21S00791 | chr10 | 118800000 | 135500000 | 2.123 | dup |
| Z21S00791 | chr11 | 200000 | 50100000 | 1.953 | del |
| Z21S00791 | chr11 | 54800000 | 135000000 | 2.067 | dup |
| Z21S00791 | chr12 | 6200000 | 30400000 | 1.929 | del |
| Z21S00791 | chr13 | 19400000 | 115100000 | 2.038 | dup |
| Z21S00791 | chr16 | 58000000 | 90200000 | 1.94 | del |
| Z21S00791 | chr17 | 25300000 | 40500000 | 1.939 | del |
| Z21S00791 | chr17 | 40500000 | 81195210 | 2.094 | dup |
| Z21S00791 | chr20 | 0 | 26300000 | 1.943 | del |
| Z21S00791 | chr20 | 29800000 | 62900000 | 2.041 | dup |
| Z21S00791 | chr22 | 22100000 | 51200000 | 1.919 | del |
| Z21S02532 | chr3 | 93500000 | 192900000 | 2.065 | dup |
| Z21S02532 | chr4 | 0 | 40200000 | 2.044 | dup |
| Z21S02532 | chr5 | 0 | 46400000 | 2.052 | dup |
| Z21S02532 | chr5 | 146000000 | 180700000 | 2.031 | dup |
| Z21S02532 | chr6 | 135100000 | 170900000 | 1.97 | del |
| Z21S02532 | chr9 | 200000 | 38800000 | 2.039 | dup |
| Z21S02532 | chr12 | 24300000 | 28800000 | 2.709 | dup |
| Z21S02532 | chr17 | 25300000 | 46000000 | 1.946 | del |
| Z21S02532 | chr18 | 18500000 | 78000000 | 2.043 | dup |
| Z21S02532 | chr20 | 29800000 | 62900000 | 2.064 | dup |
| Z21S02532 | chr21 | 15400000 | 48100000 | 1.969 | del |
| Z21S02548 | chr1 | 144800000 | 249200000 | 2.184 | dup |
| Z21S02548 | chr2 | 95500000 | 102900000 | 2.14 | dup |
| Z21S02548 | chr3 | 100000 | 90500000 | 2.168 | dup |
| Z21S02548 | chr3 | 93500000 | 126900000 | 2.161 | dup |
| Z21S02548 | chr3 | 126900000 | 197900000 | 2.358 | dup |
| Z21S02548 | chr7 | 0 | 58000000 | 2.173 | dup |
| Z21S02548 | chr7 | 61500000 | 159100000 | 2.176 | dup |
| Z21S02548 | chr8 | 100000 | 43800000 | 2.321 | dup |
| Z21S02548 | chr8 | 46900000 | 146300000 | 2.315 | dup |
| Z21S02548 | chr9 | 200000 | 38800000 | 2.328 | dup |
| Z21S02548 | chr9 | 71000000 | 141100000 | 2.343 | dup |
| Z21S02548 | chr14 | 20200000 | 107300000 | 2.161 | dup |
| Z21S03128 | chr1 | 3500000 | 6400000 | 2.209 | dup |
| Z21S03128 | chr1 | 8800000 | 12800000 | 1.847 | del |
| Z21S03128 | chr1 | 19200000 | 29700000 | 1.869 | del |
| Z21S03128 | chr1 | 37900000 | 49000000 | 1.888 | del |
| Z21S03128 | chr1 | 50400000 | 55700000 | 1.858 | del |
| Z21S03128 | chr1 | 56700000 | 59800000 | 1.793 | del |
| Z21S03128 | chr1 | 60400000 | 66100000 | 1.907 | del |
| Z21S03128 | chr1 | 68700000 | 78000000 | 2.063 | dup |
| Z21S03128 | chr1 | 144800000 | 152000000 | 1.776 | del |
| Z21S03128 | chr1 | 153600000 | 156500000 | 1.788 | del |
| Z21S03128 | chr1 | 196200000 | 198300000 | 1.741 | del |
| Z21S03128 | chr1 | 219100000 | 234500000 | 1.954 | del |
| Z21S03128 | chr2 | 0 | 6900000 | 2.194 | dup |
| Z21S03128 | chr2 | 42300000 | 44900000 | 1.763 | del |
| Z21S03128 | chr2 | 79200000 | 81700000 | 2.222 | dup |
| Z21S03128 | chr2 | 154600000 | 172200000 | 1.961 | del |
| Z21S03128 | chr2 | 220500000 | 223200000 | 2.199 | dup |
| Z21S03128 | chr3 | 2200000 | 9400000 | 2.081 | dup |
| Z21S03128 | chr3 | 74300000 | 90500000 | 2.072 | dup |
| Z21S03128 | chr4 | 0 | 3600000 | 1.856 | del |
| Z21S03128 | chr4 | 6500000 | 21700000 | 2.078 | dup |
| Z21S03128 | chr4 | 22300000 | 37400000 | 2.049 | dup |
| Z21S03128 | chr4 | 37400000 | 41500000 | 1.761 | del |
| Z21S03128 | chr4 | 41500000 | 47900000 | 2.081 | dup |
| Z21S03128 | chr4 | 52700000 | 69400000 | 2.052 | dup |
| Z21S03128 | chr4 | 128500000 | 151300000 | 2.04 | dup |
| Z21S03128 | chr4 | 160300000 | 169900000 | 2.075 | dup |
| Z21S03128 | chr4 | 170900000 | 182300000 | 2.081 | dup |
| Z21S03128 | chr5 | 0 | 8900000 | 2.197 | dup |
| Z21S03128 | chr5 | 16400000 | 36100000 | 2.041 | dup |
| Z21S03128 | chr5 | 160900000 | 168200000 | 2.079 | dup |
| Z21S03128 | chr6 | 20000000 | 40000000 | 1.933 | del |
| Z21S03128 | chr6 | 61900000 | 86400000 | 2.036 | dup |
| Z21S03128 | chr6 | 128900000 | 143100000 | 1.941 | del |
| Z21S03128 | chr7 | 51600000 | 54900000 | 2.225 | dup |
| Z21S03128 | chr7 | 98500000 | 101000000 | 1.73 | del |
| Z21S03128 | chr8 | 100000 | 8700000 | 2.111 | dup |
| Z21S03128 | chr8 | 33600000 | 37100000 | 2.221 | dup |
| Z21S03128 | chr8 | 48100000 | 67300000 | 2.062 | dup |
| Z21S03128 | chr8 | 73700000 | 97400000 | 2.032 | dup |
| Z21S03128 | chr8 | 97400000 | 100600000 | 1.84 | del |
| Z21S03128 | chr8 | 126700000 | 135900000 | 2.06 | dup |
| Z21S03128 | chr8 | 135900000 | 138500000 | 2.208 | dup |
| Z21S03128 | chr9 | 7100000 | 33000000 | 2.062 | dup |
| Z21S03128 | chr9 | 33000000 | 35900000 | 1.787 | del |
| Z21S03128 | chr9 | 113000000 | 117900000 | 1.888 | del |
| Z21S03128 | chr9 | 119500000 | 123100000 | 2.171 | dup |
| Z21S03128 | chr9 | 123100000 | 136500000 | 1.887 | del |
| Z21S03128 | chr10 | 42400000 | 51000000 | 2.1 | dup |
| Z21S03128 | chr10 | 74000000 | 77900000 | 1.737 | del |
| Z21S03128 | chr10 | 82300000 | 88100000 | 2.162 | dup |
| Z21S03128 | chr10 | 88100000 | 91600000 | 1.833 | del |
| Z21S03128 | chr10 | 101000000 | 106100000 | 1.876 | del |
| Z21S03128 | chr10 | 106100000 | 110700000 | 2.181 | dup |
| Z21S03128 | chr10 | 113800000 | 116700000 | 1.804 | del |
| Z21S03128 | chr10 | 116700000 | 123500000 | 2.08 | dup |
| Z21S03128 | chr10 | 124300000 | 132200000 | 2.087 | dup |
| Z21S03128 | chr11 | 20500000 | 32600000 | 2.069 | dup |
| Z21S03128 | chr11 | 35900000 | 45800000 | 2.115 | dup |
| Z21S03128 | chr11 | 62200000 | 78300000 | 1.865 | del |
| Z21S03128 | chr11 | 87100000 | 111400000 | 2.051 | dup |
| Z21S03128 | chr11 | 131000000 | 134900000 | 2.213 | dup |
| Z21S03128 | chr12 | 120000000 | 126200000 | 1.836 | del |
| Z21S03128 | chr12 | 126200000 | 132200000 | 2.197 | dup |
| Z21S03128 | chr13 | 98800000 | 101200000 | 1.768 | del |
| Z21S03128 | chr13 | 101200000 | 115100000 | 2.047 | dup |
| Z21S03128 | chr14 | 66800000 | 69300000 | 1.733 | del |
| Z21S03128 | chr14 | 80800000 | 87500000 | 2.089 | dup |
| Z21S03128 | chr14 | 97400000 | 99800000 | 2.239 | dup |
| Z21S03128 | chr15 | 20500000 | 29600000 | 2.137 | dup |
| Z21S03128 | chr15 | 29600000 | 46000000 | 1.928 | del |
| Z21S03128 | chr15 | 51100000 | 58400000 | 1.905 | del |
| Z21S03128 | chr15 | 65000000 | 68600000 | 1.85 | del |
| Z21S03128 | chr15 | 69400000 | 84800000 | 1.934 | del |
| Z21S03128 | chr15 | 86400000 | 102400000 | 2.04 | dup |
| Z21S03128 | chr16 | 15500000 | 18200000 | 3.097 | dup |
| Z21S03128 | chr16 | 59600000 | 66500000 | 2.167 | dup |
| Z21S03128 | chr16 | 66500000 | 73300000 | 1.821 | del |
| Z21S03128 | chr17 | 25300000 | 31300000 | 1.856 | del |
| Z21S03128 | chr17 | 54900000 | 66500000 | 1.871 | del |
| Z21S03128 | chr18 | 18500000 | 21900000 | 1.829 | del |
| Z21S03128 | chr18 | 35600000 | 46000000 | 2.051 | dup |
| Z21S03128 | chr18 | 60700000 | 78000000 | 2.039 | dup |
| Z21S03128 | chr19 | 200000 | 11600000 | 1.805 | del |
| Z21S03128 | chr20 | 29800000 | 37700000 | 1.896 | del |
| Z21S03128 | chr20 | 52500000 | 62900000 | 2.082 | dup |
| Z21S03128 | chr22 | 24100000 | 34600000 | 1.864 | del |
| Z21S03128 | chr22 | 36700000 | 44500000 | 1.894 | del |
| Z21S03697 | chr1 | 144800000 | 249200000 | 2.573 | dup |
| Z21S03697 | chr5 | 0 | 43900000 | 2.556 | dup |
| Z21S03697 | chr5 | 43900000 | 46400000 | 2.317 | dup |
| Z21S03697 | chr5 | 49500000 | 180700000 | 2.278 | dup |
| Z21S03697 | chr11 | 84200000 | 118300000 | 1.711 | del |
| Z21S03697 | chr11 | 119300000 | 135000000 | 1.698 | del |
| Z21S03697 | chr16 | 5300000 | 35300000 | 2.291 | dup |
| Z21S03697 | chr16 | 46500000 | 90200000 | 1.717 | del |
| Z21S03698 | chr5 | 0 | 46400000 | 2.047 | dup |
| Z21S03698 | chr9 | 200000 | 38800000 | 2.037 | dup |
| Z21S03698 | chr11 | 36000000 | 51400000 | 2.107 | dup |
| Z21S03698 | chr18 | 18500000 | 78000000 | 2.036 | dup |
| Z21S03693 | chr5 | 0 | 46400000 | 2.041 | dup |
| Z21S03692 | chr1 | 800000 | 55800000 | 2.073 | dup |
| Z21S03692 | chr1 | 55800000 | 92300000 | 1.956 | del |
| Z21S03692 | chr1 | 92300000 | 121300000 | 2.033 | dup |
| Z21S03692 | chr1 | 144800000 | 249200000 | 2.034 | dup |
| Z21S03692 | chr2 | 0 | 90200000 | 2.049 | dup |
| Z21S03692 | chr2 | 95500000 | 243100000 | 2.041 | dup |
| Z21S03692 | chr3 | 100000 | 56100000 | 2.066 | dup |
| Z21S03692 | chr3 | 56100000 | 90500000 | 1.967 | del |
| Z21S03692 | chr3 | 93500000 | 197900000 | 2.051 | dup |
| Z21S03692 | chr4 | 52700000 | 80800000 | 2.052 | dup |
| Z21S03692 | chr4 | 80800000 | 190900000 | 1.963 | del |
| Z21S03692 | chr5 | 0 | 46400000 | 2.045 | dup |
| Z21S03692 | chr5 | 49500000 | 67500000 | 2.033 | dup |
| Z21S03692 | chr5 | 67500000 | 180700000 | 1.958 | del |
| Z21S03692 | chr6 | 100000 | 58700000 | 1.939 | del |
| Z21S03692 | chr6 | 61900000 | 170900000 | 2.033 | dup |
| Z21S03692 | chr7 | 0 | 58000000 | 2.047 | dup |
| Z21S03692 | chr7 | 61500000 | 159100000 | 2.047 | dup |
| Z21S03692 | chr8 | 100000 | 43800000 | 1.965 | del |
| Z21S03692 | chr8 | 76700000 | 98100000 | 2.29 | dup |
| Z21S03692 | chr8 | 99500000 | 113400000 | 2.321 | dup |
| Z21S03692 | chr8 | 113400000 | 146300000 | 2.144 | dup |
| Z21S03692 | chr9 | 200000 | 38800000 | 2.136 | dup |
| Z21S03692 | chr9 | 71000000 | 141100000 | 2.033 | dup |
| Z21S03692 | chr11 | 200000 | 13400000 | 1.924 | del |
| Z21S03692 | chr11 | 68400000 | 81100000 | 2.164 | dup |
| Z21S03692 | chr11 | 81100000 | 135000000 | 1.97 | del |
| Z21S03692 | chr12 | 38000000 | 133851895 | 2.035 | dup |
| Z21S03692 | chr13 | 19400000 | 115100000 | 1.949 | del |
| Z21S03692 | chr14 | 20200000 | 107300000 | 2.041 | dup |
| Z21S03692 | chr15 | 20500000 | 92200000 | 2.098 | dup |
| Z21S03692 | chr15 | 92200000 | 102400000 | 2.248 | dup |
| Z21S03692 | chr20 | 0 | 26300000 | 2.033 | dup |
| Z21S03692 | chr20 | 29800000 | 59600000 | 2.036 | dup |
| Z21S03692 | chr21 | 15400000 | 48100000 | 1.942 | del |
| Z21S02269 | chr20 | 0 | 26300000 | 2.036 | dup |
| Z21S02269 | chr20 | 29800000 | 62900000 | 2.041 | dup |
| ZL22N0032 | chr1 | 144800000 | 176900000 | 1.969 | del |
| ZL22N0032 | chr1 | 177900000 | 249200000 | 1.97 | del |
| ZL22N0031 | chr7 | 0 | 58000000 | 2.073 | dup |
| ZL22N0031 | chr7 | 62500000 | 159100000 | 2.077 | dup |
| ZL22N0031 | chr8 | 100000 | 43800000 | 2.065 | dup |
| ZL22N0031 | chr8 | 46900000 | 146300000 | 2.075 | dup |
| ZL22N0031 | chr10 | 100000 | 39200000 | 2.06 | dup |
| ZL22N0031 | chr10 | 42400000 | 135500000 | 2.076 | dup |
| ZL22N0036 | chr1 | 800000 | 11800000 | 2.111 | dup |
| ZL22N0036 | chr1 | 11800000 | 35800000 | 1.849 | del |
| ZL22N0036 | chr1 | 35800000 | 121300000 | 2.06 | dup |
| ZL22N0036 | chr1 | 144800000 | 208200000 | 2.063 | dup |
| ZL22N0036 | chr1 | 208200000 | 249200000 | 2.324 | dup |
| ZL22N0036 | chr2 | 0 | 90200000 | 2.105 | dup |
| ZL22N0036 | chr2 | 95500000 | 243100000 | 2.046 | dup |
| ZL22N0036 | chr3 | 93500000 | 167600000 | 2.071 | dup |
| ZL22N0036 | chr3 | 167600000 | 197900000 | 2.315 | dup |
| ZL22N0036 | chr4 | 0 | 26900000 | 1.842 | del |
| ZL22N0036 | chr4 | 26900000 | 31100000 | 2.295 | dup |
| ZL22N0036 | chr4 | 31100000 | 49100000 | 2.046 | dup |
| ZL22N0036 | chr4 | 52700000 | 80800000 | 2.069 | dup |
| ZL22N0036 | chr4 | 80800000 | 144200000 | 1.816 | del |
| ZL22N0036 | chr4 | 144200000 | 179500000 | 2.055 | dup |
| ZL22N0036 | chr4 | 179500000 | 190900000 | 1.824 | del |
| ZL22N0036 | chr5 | 0 | 29800000 | 2.076 | dup |
| ZL22N0036 | chr5 | 32600000 | 46400000 | 2.067 | dup |
| ZL22N0036 | chr5 | 50300000 | 74900000 | 1.822 | del |
| ZL22N0036 | chr5 | 74900000 | 180700000 | 2.066 | dup |
| ZL22N0036 | chr6 | 100000 | 32900000 | 2.425 | dup |
| ZL22N0036 | chr6 | 32900000 | 49300000 | 2.271 | dup |
| ZL22N0036 | chr6 | 61900000 | 117300000 | 2.071 | dup |
| ZL22N0036 | chr6 | 117300000 | 170900000 | 1.819 | del |
| ZL22N0036 | chr7 | 0 | 28800000 | 1.82 | del |
| ZL22N0036 | chr7 | 28800000 | 58000000 | 2.076 | dup |
| ZL22N0036 | chr7 | 61500000 | 67600000 | 2.25 | dup |
| ZL22N0036 | chr7 | 67600000 | 86700000 | 2.416 | dup |
| ZL22N0036 | chr7 | 86700000 | 159100000 | 2.217 | dup |
| ZL22N0036 | chr8 | 3200000 | 35600000 | 1.823 | del |
| ZL22N0036 | chr8 | 37400000 | 43800000 | 2.124 | dup |
| ZL22N0036 | chr8 | 46900000 | 58000000 | 2.322 | dup |
| ZL22N0036 | chr8 | 58000000 | 60900000 | 2.549 | dup |
| ZL22N0036 | chr8 | 60900000 | 63100000 | 2.325 | dup |
| ZL22N0036 | chr8 | 63100000 | 93700000 | 2.135 | dup |
| ZL22N0036 | chr8 | 93700000 | 146300000 | 2.376 | dup |
| ZL22N0036 | chr9 | 200000 | 38800000 | 2.04 | dup |
| ZL22N0036 | chr9 | 71000000 | 95200000 | 2.039 | dup |
| ZL22N0036 | chr9 | 95200000 | 141100000 | 1.845 | del |
| ZL22N0036 | chr10 | 100000 | 4400000 | 2.306 | dup |
| ZL22N0036 | chr10 | 15400000 | 20800000 | 1.805 | del |
| ZL22N0036 | chr10 | 27500000 | 39200000 | 2.301 | dup |
| ZL22N0036 | chr10 | 42400000 | 135500000 | 2.089 | dup |
| ZL22N0036 | chr11 | 200000 | 40900000 | 1.832 | del |
| ZL22N0036 | chr11 | 41000000 | 43300000 | 2.342 | dup |
| ZL22N0036 | chr11 | 54800000 | 135000000 | 2.105 | dup |
| ZL22N0036 | chr12 | 100000 | 4400000 | 2.296 | dup |
| ZL22N0036 | chr12 | 4400000 | 34900000 | 2.041 | dup |
| ZL22N0036 | chr12 | 38000000 | 71400000 | 2.066 | dup |
| ZL22N0036 | chr12 | 71400000 | 108700000 | 1.81 | del |
| ZL22N0036 | chr12 | 108700000 | 123100000 | 2.072 | dup |
| ZL22N0036 | chr12 | 123100000 | 133851895 | 1.845 | del |
| ZL22N0036 | chr13 | 19400000 | 32500000 | 1.862 | del |
| ZL22N0036 | chr13 | 40100000 | 50000000 | 1.835 | del |
| ZL22N0036 | chr13 | 50000000 | 115100000 | 2.062 | dup |
| ZL22N0036 | chr14 | 20200000 | 107300000 | 2.06 | dup |
| ZL22N0036 | chr15 | 20500000 | 102400000 | 2.12 | dup |
| ZL22N0036 | chr16 | 46500000 | 90200000 | 1.839 | del |
| ZL22N0036 | chr17 | 0 | 22300000 | 1.84 | del |
| ZL22N0036 | chr17 | 25300000 | 43500000 | 1.816 | del |
| ZL22N0036 | chr17 | 43700000 | 81195210 | 2.098 | dup |
| ZL22N0036 | chr18 | 6300000 | 15000000 | 1.82 | del |
| ZL22N0036 | chr18 | 18500000 | 78000000 | 1.828 | del |
| ZL22N0036 | chr20 | 0 | 26300000 | 2.075 | dup |
| ZL22N0036 | chr20 | 29800000 | 62900000 | 2.079 | dup |
| ZL22N0036 | chr21 | 15400000 | 48100000 | 2.059 | dup |
| ZL22N0036 | chr22 | 21900000 | 51200000 | 1.851 | del |
| ZL22N0035 | chr1 | 800000 | 31000000 | 1.73 | del |
| ZL22N0035 | chr1 | 31000000 | 116200000 | 2.205 | dup |
| ZL22N0035 | chr1 | 116200000 | 119900000 | 1.697 | del |
| ZL22N0035 | chr1 | 150000000 | 152100000 | 2.649 | dup |
| ZL22N0035 | chr1 | 156900000 | 168000000 | 2.219 | dup |
| ZL22N0035 | chr1 | 168000000 | 173000000 | 2.71 | dup |
| ZL22N0035 | chr1 | 173000000 | 249200000 | 2.193 | dup |
| ZL22N0035 | chr2 | 0 | 26400000 | 1.713 | del |
| ZL22N0035 | chr2 | 63800000 | 70600000 | 2.483 | dup |
| ZL22N0035 | chr2 | 70600000 | 79100000 | 2.721 | dup |
| ZL22N0035 | chr2 | 79100000 | 90200000 | 2.222 | dup |
| ZL22N0035 | chr2 | 95500000 | 205400000 | 2.21 | dup |
| ZL22N0035 | chr2 | 205400000 | 242300000 | 1.703 | del |
| ZL22N0035 | chr3 | 100000 | 54700000 | 1.964 | del |
| ZL22N0035 | chr3 | 54700000 | 90500000 | 2.21 | dup |
| ZL22N0035 | chr3 | 93500000 | 97100000 | 2.218 | dup |
| ZL22N0035 | chr3 | 97100000 | 116100000 | 2.741 | dup |
| ZL22N0035 | chr3 | 116700000 | 128900000 | 2.691 | dup |
| ZL22N0035 | chr3 | 129600000 | 136900000 | 3.171 | dup |
| ZL22N0035 | chr3 | 137700000 | 157900000 | 3.197 | dup |
| ZL22N0035 | chr3 | 157900000 | 171100000 | 3.695 | dup |
| ZL22N0035 | chr3 | 171100000 | 183900000 | 3.211 | dup |
| ZL22N0035 | chr3 | 183900000 | 197900000 | 3.453 | dup |
| ZL22N0035 | chr4 | 0 | 9000000 | 2.716 | dup |
| ZL22N0035 | chr4 | 9700000 | 49100000 | 2.227 | dup |
| ZL22N0035 | chr4 | 52700000 | 78400000 | 2.215 | dup |
| ZL22N0035 | chr4 | 78400000 | 190900000 | 1.683 | del |
| ZL22N0035 | chr5 | 0 | 46400000 | 2.244 | dup |
| ZL22N0035 | chr5 | 49500000 | 57800000 | 2.209 | dup |
| ZL22N0035 | chr5 | 62700000 | 166400000 | 1.693 | del |
| ZL22N0035 | chr5 | 167600000 | 170200000 | 1.732 | del |
| ZL22N0035 | chr5 | 170200000 | 180700000 | 2.218 | dup |
| ZL22N0035 | chr6 | 2600000 | 17600000 | 2.444 | dup |
| ZL22N0035 | chr6 | 17600000 | 25900000 | 2.214 | dup |
| ZL22N0035 | chr6 | 25900000 | 30500000 | 1.703 | del |
| ZL22N0035 | chr6 | 61900000 | 170900000 | 1.69 | del |
| ZL22N0035 | chr7 | 1900000 | 38200000 | 1.957 | del |
| ZL22N0035 | chr7 | 38200000 | 51700000 | 1.701 | del |
| ZL22N0035 | chr7 | 62500000 | 65800000 | 2.239 | dup |
| ZL22N0035 | chr7 | 65800000 | 159100000 | 2.481 | dup |
| ZL22N0035 | chr8 | 100000 | 6500000 | 2.256 | dup |
| ZL22N0035 | chr8 | 6500000 | 36100000 | 1.711 | del |
| ZL22N0035 | chr8 | 36100000 | 43800000 | 2.246 | dup |
| ZL22N0035 | chr8 | 46900000 | 95800000 | 2.238 | dup |
| ZL22N0035 | chr8 | 95800000 | 107800000 | 2.968 | dup |
| ZL22N0035 | chr8 | 107800000 | 118000000 | 3.511 | dup |
| ZL22N0035 | chr8 | 118800000 | 122400000 | 3.516 | dup |
| ZL22N0035 | chr8 | 122400000 | 128800000 | 3.021 | dup |
| ZL22N0035 | chr8 | 129700000 | 144200000 | 3.052 | dup |
| ZL22N0035 | chr9 | 200000 | 4200000 | 1.173 | del |
| ZL22N0035 | chr9 | 4200000 | 11200000 | 1.427 | del |
| ZL22N0035 | chr9 | 74100000 | 83600000 | 2.489 | dup |
| ZL22N0035 | chr9 | 83600000 | 138200000 | 2.188 | dup |
| ZL22N0035 | chr9 | 138200000 | 141100000 | 1.756 | del |
| ZL22N0035 | chr10 | 100000 | 39200000 | 1.689 | del |
| ZL22N0035 | chr10 | 42400000 | 135500000 | 1.97 | del |
| ZL22N0035 | chr11 | 200000 | 7300000 | 1.72 | del |
| ZL22N0035 | chr11 | 7300000 | 28700000 | 1.948 | del |
| ZL22N0035 | chr11 | 28700000 | 51400000 | 2.24 | dup |
| ZL22N0035 | chr11 | 54800000 | 77400000 | 2.223 | dup |
| ZL22N0035 | chr11 | 78500000 | 135000000 | 2.236 | dup |
| ZL22N0035 | chr12 | 100000 | 34900000 | 2.193 | dup |
| ZL22N0035 | chr12 | 38000000 | 42100000 | 2.195 | dup |
| ZL22N0035 | chr12 | 42100000 | 66100000 | 1.69 | del |
| ZL22N0035 | chr12 | 66100000 | 90100000 | 2.206 | dup |
| ZL22N0035 | chr12 | 90100000 | 93400000 | 2.49 | dup |
| ZL22N0035 | chr12 | 93400000 | 112000000 | 1.935 | del |
| ZL22N0035 | chr12 | 112000000 | 133851895 | 2.204 | dup |
| ZL22N0035 | chr13 | 19400000 | 44100000 | 2.184 | dup |
| ZL22N0035 | chr13 | 44100000 | 79000000 | 1.671 | del |
| ZL22N0035 | chr14 | 20200000 | 101200000 | 2.206 | dup |
| ZL22N0035 | chr14 | 101200000 | 107300000 | 1.758 | del |
| ZL22N0035 | chr15 | 20500000 | 26000000 | 2.233 | dup |
| ZL22N0035 | chr15 | 26000000 | 71700000 | 1.696 | del |
| ZL22N0035 | chr15 | 71700000 | 80700000 | 2.192 | dup |
| ZL22N0035 | chr15 | 80700000 | 102400000 | 2.452 | dup |
| ZL22N0035 | chr16 | 100000 | 35300000 | 2.219 | dup |
| ZL22N0035 | chr16 | 46500000 | 52500000 | 2.231 | dup |
| ZL22N0035 | chr16 | 52500000 | 73400000 | 1.716 | del |
| ZL22N0035 | chr16 | 75100000 | 84000000 | 2.732 | dup |
| ZL22N0035 | chr16 | 84000000 | 90200000 | 2.527 | dup |
| ZL22N0035 | chr17 | 0 | 22300000 | 1.707 | del |
| ZL22N0035 | chr17 | 25300000 | 46000000 | 1.703 | del |
| ZL22N0035 | chr18 | 100000 | 15000000 | 2.23 | dup |
| ZL22N0035 | chr18 | 18500000 | 30600000 | 2.218 | dup |
| ZL22N0035 | chr18 | 30600000 | 78000000 | 1.689 | del |
| ZL22N0035 | chr19 | 46300000 | 59100000 | 1.709 | del |
| ZL22N0035 | chr20 | 1200000 | 25100000 | 3.026 | dup |
| ZL22N0035 | chr20 | 29800000 | 32000000 | 2.261 | dup |
| ZL22N0035 | chr20 | 32000000 | 41200000 | 1.718 | del |
| ZL22N0035 | chr20 | 43000000 | 58600000 | 2.461 | dup |
| ZL22N0035 | chr20 | 58600000 | 62900000 | 3.066 | dup |
| ZL22N0035 | chr21 | 15400000 | 23600000 | 2.31 | dup |
| ZL22N0035 | chr21 | 23600000 | 48100000 | 1.7 | del |
| ZL22N0035 | chr22 | 35900000 | 38800000 | 1.489 | del |
| ZL22N0002 | chr3 | 93500000 | 197900000 | 2.041 | dup |
| ZL22N0002 | chr5 | 49500000 | 180700000 | 1.97 | del |
| ZL22N0002 | chr6 | 61900000 | 170900000 | 1.967 | del |
| ZL22N0002 | chr7 | 61500000 | 159100000 | 2.032 | dup |
| ZL22N0002 | chr9 | 200000 | 38800000 | 2.032 | dup |
| ZL22N0002 | chr12 | 100000 | 21800000 | 2.084 | dup |
| ZL22N0002 | chr12 | 21800000 | 25700000 | 2.529 | dup |
| ZL22N0002 | chr12 | 38000000 | 133851895 | 2.037 | dup |
| ZL22N0002 | chr20 | 29800000 | 61800000 | 2.053 | dup |
| Z21S02544 | chr1 | 800000 | 47900000 | 1.933 | del |
| Z21S02544 | chr1 | 48800000 | 95900000 | 1.939 | del |
| Z21S02544 | chr1 | 95900000 | 121300000 | 2.047 | dup |
| Z21S02544 | chr1 | 155100000 | 227600000 | 1.929 | del |
| Z21S02544 | chr2 | 0 | 13600000 | 1.908 | del |
| Z21S02544 | chr2 | 13600000 | 90200000 | 2.035 | dup |
| Z21S02544 | chr2 | 151400000 | 194700000 | 2.101 | dup |
| Z21S02544 | chr2 | 223500000 | 243100000 | 1.907 | del |
| Z21S02544 | chr3 | 44700000 | 90500000 | 1.904 | del |
| Z21S02544 | chr3 | 93500000 | 143700000 | 1.93 | del |
| Z21S02544 | chr3 | 143700000 | 149600000 | 2.16 | dup |
| Z21S02544 | chr3 | 149600000 | 152700000 | 2.446 | dup |
| Z21S02544 | chr3 | 152700000 | 156100000 | 2.185 | dup |
| Z21S02544 | chr3 | 160400000 | 170200000 | 2.199 | dup |
| Z21S02544 | chr3 | 170200000 | 172700000 | 2.414 | dup |
| Z21S02544 | chr3 | 177900000 | 182300000 | 2.353 | dup |
| Z21S02544 | chr3 | 182300000 | 197900000 | 2.111 | dup |
| Z21S02544 | chr4 | 0 | 27000000 | 1.935 | del |
| Z21S02544 | chr4 | 52700000 | 66100000 | 2.047 | dup |
| Z21S02544 | chr4 | 66100000 | 68500000 | 2.278 | dup |
| Z21S02544 | chr4 | 98000000 | 122000000 | 1.801 | del |
| Z21S02544 | chr4 | 122000000 | 146900000 | 1.911 | del |
| Z21S02544 | chr4 | 146900000 | 163600000 | 2.229 | dup |
| Z21S02544 | chr4 | 163600000 | 190900000 | 2.366 | dup |
| Z21S02544 | chr5 | 0 | 14500000 | 2.459 | dup |
| Z21S02544 | chr5 | 16500000 | 46400000 | 2.368 | dup |
| Z21S02544 | chr5 | 52600000 | 125100000 | 1.812 | del |
| Z21S02544 | chr5 | 125100000 | 143000000 | 1.964 | del |
| Z21S02544 | chr5 | 143000000 | 155800000 | 2.068 | dup |
| Z21S02544 | chr5 | 155800000 | 180700000 | 1.898 | del |
| Z21S02544 | chr6 | 100000 | 17600000 | 2.135 | dup |
| Z21S02544 | chr6 | 17600000 | 47700000 | 1.917 | del |
| Z21S02544 | chr6 | 72400000 | 170900000 | 1.923 | del |
| Z21S02544 | chr7 | 0 | 45100000 | 1.84 | del |
| Z21S02544 | chr7 | 66800000 | 137800000 | 2.11 | dup |
| Z21S02544 | chr7 | 137800000 | 159100000 | 1.963 | del |
| Z21S02544 | chr8 | 100000 | 43800000 | 1.942 | del |
| Z21S02544 | chr8 | 46900000 | 74200000 | 2.107 | dup |
| Z21S02544 | chr8 | 74200000 | 124200000 | 2.214 | dup |
| Z21S02544 | chr8 | 125700000 | 146300000 | 2.274 | dup |
| Z21S02544 | chr9 | 200000 | 38800000 | 2.036 | dup |
| Z21S02544 | chr9 | 71000000 | 107400000 | 2.034 | dup |
| Z21S02544 | chr9 | 111600000 | 122600000 | 2.265 | dup |
| Z21S02544 | chr9 | 122600000 | 141100000 | 1.942 | del |
| Z21S02544 | chr10 | 43800000 | 135500000 | 1.945 | del |
| Z21S02544 | chr11 | 200000 | 11600000 | 1.808 | del |
| Z21S02544 | chr11 | 11600000 | 51400000 | 1.905 | del |
| Z21S02544 | chr11 | 54800000 | 79600000 | 2.07 | dup |
| Z21S02544 | chr11 | 82100000 | 135000000 | 1.936 | del |
| Z21S02544 | chr12 | 100000 | 21000000 | 1.923 | del |
| Z21S02544 | chr12 | 38000000 | 56200000 | 2.138 | dup |
| Z21S02544 | chr12 | 56200000 | 133851895 | 1.925 | del |
| Z21S02544 | chr13 | 19400000 | 100900000 | 1.814 | del |
| Z21S02544 | chr13 | 100900000 | 115100000 | 1.925 | del |
| Z21S02544 | chr14 | 23900000 | 107300000 | 1.927 | del |
| Z21S02544 | chr15 | 20500000 | 60800000 | 1.917 | del |
| Z21S02544 | chr15 | 63500000 | 102400000 | 1.894 | del |
| Z21S02544 | chr16 | 46500000 | 90200000 | 1.829 | del |
| Z21S02544 | chr17 | 0 | 22300000 | 1.918 | del |
| Z21S02544 | chr18 | 100000 | 15000000 | 1.934 | del |
| Z21S02544 | chr18 | 18500000 | 78000000 | 1.951 | del |
| Z21S02544 | chr19 | 27700000 | 43900000 | 2.222 | dup |
| Z21S02544 | chr20 | 0 | 10800000 | 2.431 | dup |
| Z21S02544 | chr20 | 10800000 | 26300000 | 2.173 | dup |
| Z21S02544 | chr21 | 20300000 | 48100000 | 1.936 | del |
| Z21S02544 | chr22 | 17200000 | 23000000 | 2.202 | dup |
| Z21S02544 | chr22 | 23700000 | 32300000 | 2.216 | dup |
| Z21S02544 | chr22 | 32300000 | 40900000 | 2.367 | dup |
| Z21S02544 | chr22 | 40900000 | 51200000 | 1.925 | del |
