# Supplementary figures and images for: Can circulating cell free DNA be a promising marker in ovarian cancer? – a genome-scale profiling study in a single institution
Source: J Ovarian Res. 2023 Jan 14;16:11. doi: 10.1186/s13048-022-01068-z (PMC9840311; doi:10.1186/s13048-022-01068-z)

A

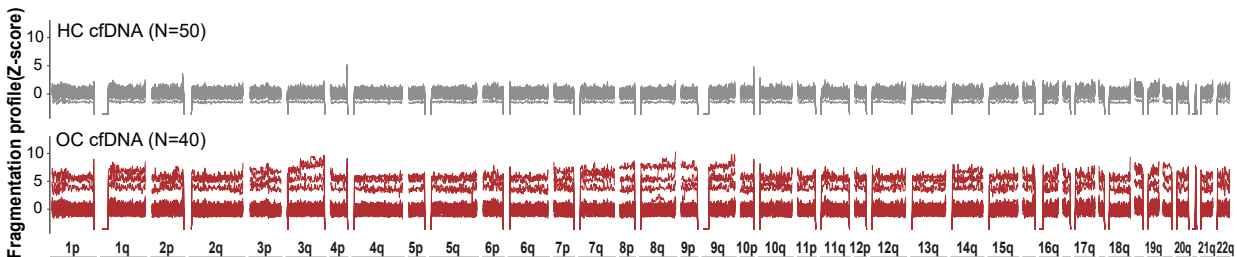

B

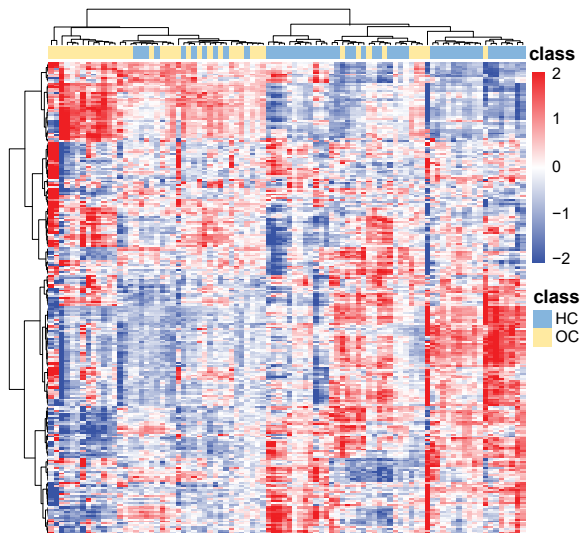

Supplement: Supplementary file 2 — Additional file 2: Supplementary Fig. 1. Genome-wide cfDNA fragmentation and end motif profiles. (A) Genome-wide cfDNA fragmentation profiles (defined as the ratio of short to long fragments) are shown in 1-Mb bins for 50 healthy individuals (top) and 40 patients with ovarian cancer (bottom). (B) Heat map classification using 256 motif. [file 13048_2022_1068_MOESM2_ESM.pdf]

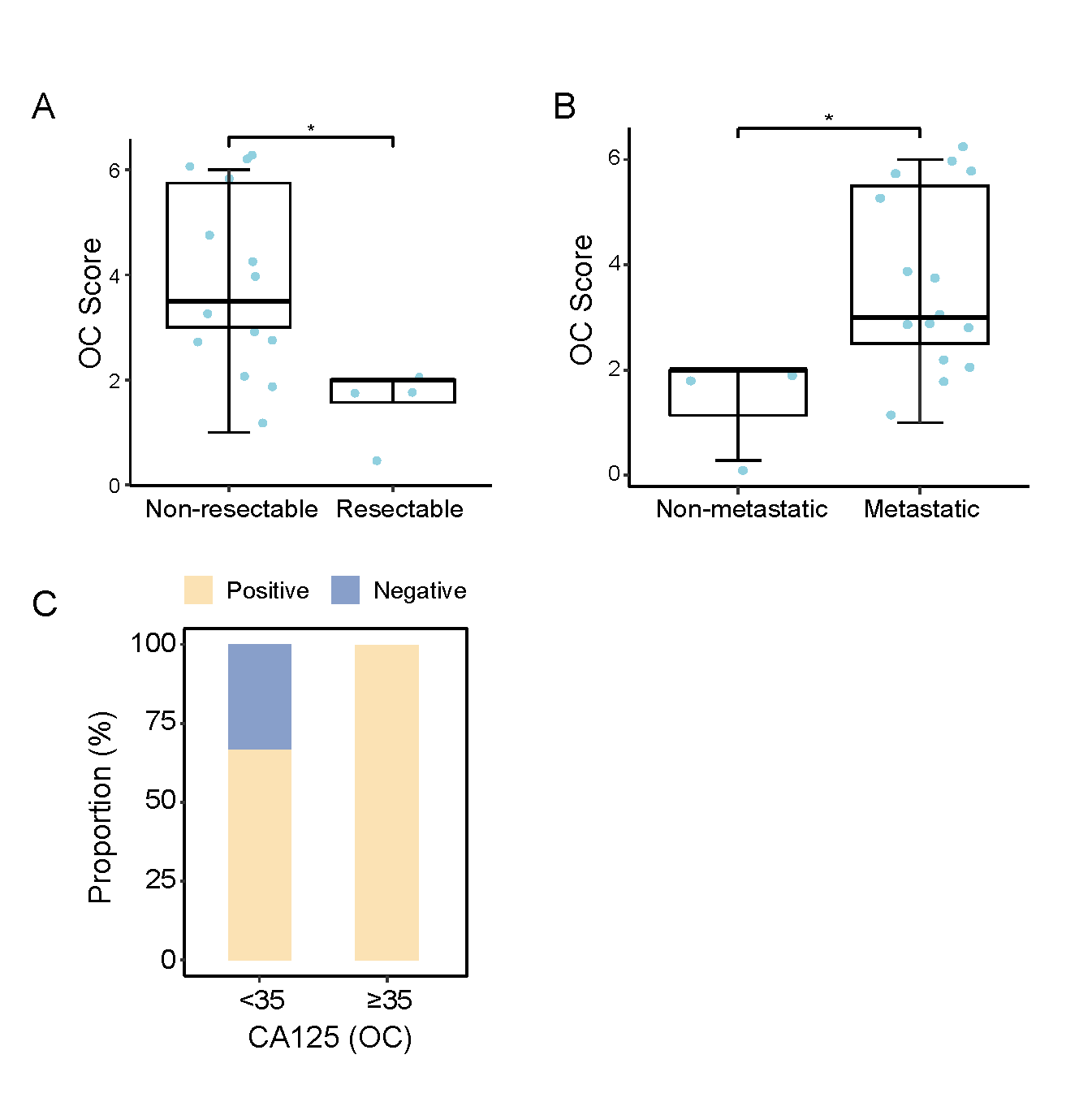

Supplement: Supplementary file 3 — Additional file 3: Supplementary Fig. 2. The detection ability of OC score in different clinical subgroups. (A-B) The ability of OC score to distinguish resectability and metastatic status. (C) Detection rate of OC score in different CA125 contents. [file 13048_2022_1068_MOESM3_ESM.tiff]
